# Advances in “Omics” Approaches for Improving Toxic Metals/Metalloids Tolerance in Plants

**DOI:** 10.3389/fpls.2021.794373

**Published:** 2022-01-04

**Authors:** Ali Raza, Javaria Tabassum, Zainab Zahid, Sidra Charagh, Shanza Bashir, Rutwik Barmukh, Rao Sohail Ahmad Khan, Fernando Barbosa, Chong Zhang, Hua Chen, Weijian Zhuang, Rajeev K. Varshney

**Affiliations:** ^1^Key Laboratory of Ministry of Education for Genetics, Breeding and Multiple Utilization of Crops, Center of Legume Crop Genetics and Systems Biology/College of Agriculture, Oil Crops Research Institute, Fujian Agriculture and Forestry University (FAFU), Fuzhou, China; ^2^State Key Laboratory of Rice Biology, China National Rice Research Institute, Chinese Academy of Agricultural Sciences (CAAS), Hangzhou, China; ^3^School of Civil and Environmental Engineering (SCEE), Institute of Environmental Sciences and Engineering (IESE), National University of Sciences and Technology (NUST), Islamabad, Pakistan; ^4^Center of Excellence in Genomics & Systems Biology, International Crops Research Institute for the Semi-Arid Tropics (ICRISAT), Hyderabad, India; ^5^Centre of Agricultural Biochemistry and Biotechnology (CABB), University of Agriculture, Faisalabad, Pakistan; ^6^Department of Clinical Analysis, Toxicology and Food Sciences, University of Sao Paulo, Ribeirão Preto, Brazil; ^7^State Agricultural Biotechnology Centre, Centre for Crop and Food Innovation, Food Futures Institute, Murdoch University, Murdoch, WA, Australia

**Keywords:** abiotic stress, CRISPR/Cas system, genomics, metabolomics, proteomics, speed breeding, miRNAomics

## Abstract

Food safety has emerged as a high-urgency matter for sustainable agricultural production. Toxic metal contamination of soil and water significantly affects agricultural productivity, which is further aggravated by extreme anthropogenic activities and modern agricultural practices, leaving food safety and human health at risk. In addition to reducing crop production, increased metals/metalloids toxicity also disturbs plants’ demand and supply equilibrium. Counterbalancing toxic metals/metalloids toxicity demands a better understanding of the complex mechanisms at physiological, biochemical, molecular, cellular, and plant level that may result in increased crop productivity. Consequently, plants have established different internal defense mechanisms to cope with the adverse effects of toxic metals/metalloids. Nevertheless, these internal defense mechanisms are not adequate to overwhelm the metals/metalloids toxicity. Plants produce several secondary messengers to trigger cell signaling, activating the numerous transcriptional responses correlated with plant defense. Therefore, the recent advances in omics approaches such as genomics, transcriptomics, proteomics, metabolomics, ionomics, miRNAomics, and phenomics have enabled the characterization of molecular regulators associated with toxic metal tolerance, which can be deployed for developing toxic metal tolerant plants. This review highlights various response strategies adopted by plants to tolerate toxic metals/metalloids toxicity, including physiological, biochemical, and molecular responses. A seven-(omics)-based design is summarized with scientific clues to reveal the stress-responsive genes, proteins, metabolites, miRNAs, trace elements, stress-inducible phenotypes, and metabolic pathways that could potentially help plants to cope up with metals/metalloids toxicity in the face of fluctuating environmental conditions. Finally, some bottlenecks and future directions have also been highlighted, which could enable sustainable agricultural production.

## Introduction

Over the last few decades, intensive anthropogenic activities and modern farming practices have led to the contamination of ecosystems by toxic metals/metalloids (Rai et al., 2019), an alarming global concern. Toxic metals/metalloids are ubiquitous in the earth’s crust and possess multiple benefits but can be harmful to the ecosystem when present in excess amounts (Gashi et al., 2020). Abiotic stresses (e.g., toxic metals/metalloids contamination, drought, salinity, etc.) are amongst some important factors affecting the growth and productivity of crop plants, resulting in up to 70% yield losses (Rai et al., 2019; Raza et al., 2020, 2021a; Roorkiwal et al., 2020; Varshney et al., 2021c). Climate changes give rise to several environmental stresses, including toxic metals/metalloids. Consequently, climate change significantly impacts the toxic metals/metalloids pollutions based on bioavailability, fate, and toxicity (Wijngaard et al., 2017; Wu et al., 2017; Oyewo et al., 2020). Among various environmental stresses, drought stress may cause an upsurge in eutrophication and toxic metals/metalloids meditations. Whereas the flooding stress may cause more toxic metals/metalloids meditations owing to desorption or re-suspension signifying that climate change determined impacts on toxic metals/metalloids transport is a composite and dynamic environmental problem, demanding a systematic understanding of toxic metals/metalloids accessibility, transport, and uptake pathways (Wijngaard et al., 2017; Wu et al., 2017; Oyewo et al., 2020).

Plants require mineral nutrients for proper growth and development. For instance, they need macro-nutrients and micro-nutrients to carry out primary functions like metabolism, photosynthesis, synthesis of enzymes, DNA and pigments, chlorophyll (Chl) functioning, nitrogen (N) fixation, etc. (Malar et al., 2016; Patel et al., 2020; Salim and Raza, 2020). Among these nutrients, some metals/metalloids such as copper (Cu), zinc (Zn), nickel (Ni), iron (Fe), selenium (Se), etc. are required in trace amounts (Karthika et al., 2018; Hasanuzzaman et al., 2020a), and can be toxic when taken up in excess by the plants (Pandey et al., 2019; Hasanuzzaman et al., 2020a). Besides, other non-essential metals/metalloid elements, like arsenic (As), cadmium (Cd), lead (Pb), mercury (Hg), are toxic to plant even at low amounts (Asati et al., 2016; Mawia et al., 2020; Raza et al., 2021a). The toxicity of non-essential toxic metals/metalloids in plants can lead to severe damages, including reduced or inhibited growth, low biomass and reduced production, chlorosis, water and nutrient imbalance, denaturation of essential enzymes and proteins, production of reactive oxygen species (ROS), disturbed electron transport chain, lipid peroxidation, and at times ultimately plant death (Rucińska-Sobkowiak, 2016; Venkatachalam et al., 2017; Jalmi et al., 2018; Tiwari and Lata, 2018; Gashi et al., 2020; Salehi et al., 2021). As a mechanism of toxicity, some non-essential toxic metals/metalloids tend to replace the essential ones in enzymes and pigments, thus disrupting their functioning (Sarwar et al., 2017; Erickson et al., 2019). Thus, plants need these metals/metalloids at a low amount to perform their metabolic activities.

Various biotechnological techniques are being implied to get an insight and in-depth understanding of the mechanisms and pathways involved in plant responses and tolerance toward toxic metals/metalloids toxicity. “Omics” approaches such as genomics (genes and complete DNA), transcriptomics (coding RNA and its types), proteomics (proteins), metabolomics (profiling of metabolites), ionomics (micro and macro ions profiling), miRNAomics (non-coding RNA), and phenomics (plant phenotype) are being widely implied for this purpose (Jamla et al., 2021a; Khan et al., 2021; Raza et al., 2021a). Molecular regulators (genes, RNA, metabolites, and proteins) and their related activities like replication, translation, post-translation, transcription, etc., play a pivotal role in the performance and maintenance of critical plant functions. Since they also determine plant responses to toxic metals/metalloids stress, the understanding of regulatory principles at the genetic level is necessary. Genomics and transcriptomics approaches hold the potential to provide insights into such complex and intricate processes during plant development (Pirzadah et al., 2019). Proteomics offers insights into the stress-inducible proteins and their involvement in mitigation against toxic metals/metalloids toxicity (Wen et al., 2019). It also provides cues into protein profiles from the cellular to organ level and offers insights into protein behavior under stress conditions (Wen et al., 2019). Metabolomics enables an understanding of the differentially regulated metabolites and complex metabolic activities occurring within the plant under diverse conditions (Razzaq et al., 2019; Raza, 2020). Furthermore, ionomics provides insights into the nutrient and trace element composition of the plant, as well as activities and mechanisms involved in uptake, storage, assimilation, and the plants’ responses to toxic metals (Stich et al., 2020). MicroRNAs (miRNAs), a group of single-stranded, non-coding micro RNAs, have been shown to regulate gene expression at a post-transcriptional level. Plants respond to stress (including toxic metals/metalloids) by triggering the miRNAs that work by cleaving or neutralizing the transcribed mRNA according to the plant needs under stressed conditions (Dubey et al., 2021; Jamla et al., 2021b).

In this review, we start by discussing the physiological, biochemical, and molecular responses of the plants toward toxic metals/metalloids toxicity. We then describe recent innovations in “omics” approaches such as genomics, transcriptomics, proteomics, metabolomics, ionomics, miRNAomics, and phenomics that could empower the development of toxic metals/metalloids tolerant plants. Further, we summarize some important online databases/tools for the integration of omics data. Additionally, an overview of some plant-based remediation approaches has also been discussed. We aim to offer a comprehensive overview of intricate plant responses to toxic metal stress and highlight the potential of omics approaches for developing toxic metals/metalloids tolerant plants in future.

## Plant Responses to Toxic Metals/Metalloids Toxicity

Since plants constitute the primary level of the food chain, harmful metals/metalloids may enter the food chain through plants, thereby posing a threat of exposure to animals and especially humans, and may cause several physiological, morphological, and metabolic disorders and abnormalities (Rai et al., 2019). Increased metals/metalloids toxicity has resulted in reduced crop and food production by disturbing the demand and supply equilibrium, demanding an urgent need to develop strategies for combating the situation and enhancing plant tolerance toward toxic metals/metalloids (Rai et al., 2019; Gashi et al., 2020).

**FIGURE 1:**
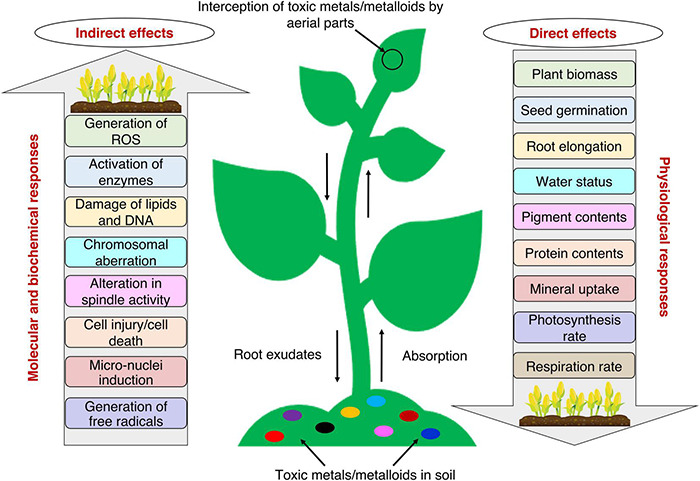
Plant responses to toxic metals/metalloids toxicity with possible direct and indirect effects on crop productivity. Plants interact with toxic metals/metalloids via above-ground and/or below-ground parts. The toxic effects of several toxic metals/metalloids decrease the physiological responses and increase the molecular and biochemical responses.

Plants being sessile organisms, have no escape when it comes to unfavorable environmental conditions. Several physiological, biochemical, and molecular responses are generated in the plant due to toxic metals/metalloids toxicity, as these toxicants interfere with several natural processes (Figure 1; Raza et al., 2021a). The interference of toxic metals/metalloids with plant processes takes place mainly through: (i) competition at the root absorption surface level with the essential metal ions for uptake; (ii) displacement of ions from specific binding sites and essential biomolecules; (iii) displacement of a critical functional group from proteins (e.g., sulfhydryl group, -SH), denaturing them and making them inactive; and (iv) the formation of ROS which interacts with and deteriorates biomolecules and disrupts the metabolic activities (Ghori et al., 2019; Mishra S. et al., 2019).

The early responses of the plant to toxic metals/metalloids stress are easier to study, whereas signal transduction at later stages of exposure is difficult to detect (Jalmi et al., 2018). Detection of stress triggers plants’ defense mechanisms through various pathways (Raza et al., 2021a). For instance, root endings detect the presence of metals and send signals from the roots throughout the plant cells and into different cell organelles (Dalvi and Bhalerao, 2013). Plants first attempt to prevent the entry of metal ions into the body by immobilizing ions with the help of their mycorrhizal associations and converting them to complexes of organic acids, amino acids, etc., secreted from the root endings (Dalvi and Bhalerao, 2013; Jalmi et al., 2018). Toxic metals/metalloids that enter the plant body may get accumulated within vacuoles and/or get bound to the cell wall and proteins, thereby causing a further modification of physiological, biochemical, and molecular activities when transported to different plant parts (Figure 1; Jalmi et al., 2018).

### Physiological Responses

Toxic metals/metalloids toxicity induces and alters many physiological responses in plants. One of the most observed plant responses is the reduction or inhibition of plant growth and development (Rucińska-Sobkowiak, 2016; Ghori et al., 2019; Raza et al., 2020, 2021a). For instance, Zn, Cd, Cu, Hg, As, Ni, chromium (Cr), and many other prominent metal toxicants have been reported to retard plant growth, seed germination and cause other morphological modifications (Ghori et al., 2019). Moreover, Pb has been reported to reduce growth in alfalfa (Hattab et al., 2016) and *Acalypha indica* (Venkatachalam et al., 2017). Increasing concentrations of Hg from 5 to 80 μg mL^–1^ decreased roots, shoots, and leaves content in *Jatropha curcas* (Marrugo-Negrete et al., 2016). Nickel has been reported to reduce seed germination in sunflower due to lower α-amylase and protease activities (Ashraf et al., 2011). Furthermore, adverse effects on leaf area due to Cd contamination were observed in tomato (Rehman et al., 2011), and production of chlorosis by Cd was reported in *Phaseolus vulgaris* (Bahmani et al., 2012). An account of the effect on pollen germination has been found in *Pisum sativum* by Cd and Cu toxicity (Sabrine et al., 2010).

Since roots represent the first plant parts to encounter toxic metals/metalloids stress in the soil, a decrease in root growth, root hair surface, and enhanced root dieback has been observed (Rucińska-Sobkowiak, 2016). Root cells tend to harden their cell walls and inhibit growth by avoiding the entrance of Cu ions in *Festuca arundinacea* and *Lolium perenne* (Zhao et al., 2010). Root cells further stimulated the entered metals/metalloids ions into the vacuole to prevent their interference with the organelle functions. For instance, Eleftheriou et al. (2015) reported Cr sequestration in the cell wall and vacuole in *Arabidopsis thaliana*. The plasma membranes in cells work in a highly controlled fashion, preventing unwanted materials from entering the organelles and mediating the harmful effects of various stresses. Despite this, Cd has been reported to disrupt the structure and functionality of the plasma membrane (Janicka-Russak et al., 2012). Such effects can further produce many undesirable outcomes, such as disruption of water and nutrient supply.

Although few metals/metalloids such as Zn, Fe, Cu, cobalt (Co), molybdenum (Mo), and manganese (Mn) are considered as essential elements for photosynthesis (Schmidt et al., 2020), their increased concentration has been shown to alter photosynthetic activities in plants (Chandra and Kang, 2016). Some metals/metalloids (e.g., Fe) act as necessary co-factors for various enzymes involved in the photosynthetic process (Balk and Schaedler, 2014); however, they tend to retard normal activities when present at higher concentrations (Asati et al., 2016). For instance, Pb and Cd were found to inhibit photosynthetic pigments in *Davidia involucrate* (Yang Y. et al., 2020). Toxic metals/metalloids toxicity also affects the overall photosynthetic apparatus and the rate of photosynthesis, causing a reduction in Chl content and mesophyll thickness (Pereira et al., 2016; Schmidt et al., 2020). In wheat, Cr has been reported to reduce the electron transport chain rate and negatively affect light-harvesting complex of photosystem II (PSII) by reducing the number of active reaction sites (Mathur et al., 2016).

Toxic metals/metalloids toxicity tends to affect the nutrient uptake, water uptake, and their translocation and assimilation in plants. Several alterations in plant functions, including dehydration due to inhibition of water translocation (Hussain et al., 2013), reduced transpiration, and altered stomatal activity, have also been observed (Wani et al., 2018). Decreased transpiration was observed for poplar hybrid in response to Cd, Cu, Cr, and Zn exposure (Chandra and Kang, 2016). In contrast, reduced stomatal activity was observed in *Pistacia vera* due to Zn toxicity (Tavallali, 2017). Moreover, xylem and phloem functionality were affected due to toxic metals/metalloids contamination, e.g., reduction in root xylem area was observed in *Salix caprea* in response to Zn and Cd exposure (Vaculík et al., 2012). Toxic ions have been found to compete with essential ions for translocation throughout the plant (Rucińska-Sobkowiak, 2016). For example, Cd was identified to compete with essential ions such as Fe, Mg, and calcium (Ca), among others (Raza et al., 2020).

### Biochemical Responses

Toxic metals/metalloids usually trigger biochemical responses by displacing/replacing essential ions or blocking functional groups. They compete with crucial nutrients at bindings sites of enzymes, rendering them denatured and inhibiting their activity (Ghori et al., 2019). For instance, the biochemical response of Cr on mung bean seeds showed a significant decline in protein, Chl, and starch content under 50% metal contaminated soil (Rath et al., 2019). Similarly, Se-stressed rice plants were found to promote Chl and protein degradation in response to metal accumulation. Alteration in these biochemical processes caused significant physiological toxicity symptoms in plants (Gouveia et al., 2020). Different ornamental plant species grown under urban Pb-contaminated soil showed a decline in their net photosynthetic rate and root activity due to stress responses. In contrast, the levels of soluble sugars, leaf proline content, and membrane stability index, altogether maintain osmotic adjustment, form metal-proline complexes and identify the extent of membrane damage, respectively, were found to increase significantly (Song et al., 2020b). The biochemical responses of kenaf (*Hibiscus cannabinus* L.) revealed Cu-induced damage in root and shoot tissues as observed through excess production of malondialdehyde (MDA), hydrogen peroxide (H_2_O_2_), and electrolyte leakage. Its negative impacts on the plant were mediated through antioxidative enzyme activities, which increase with enhancing metal concentration (Saleem et al., 2020).

The assessment of cowpea subjected to Fe toxicity showed an increase in the MDA content (Ifie et al., 2020). Another study on halophytic phytoremediator of Cu, *Sesuvium portulacastrum*, also reported similar findings where plants were affected by the toxic effects of Cu at higher concentrations. The MDA production was mediated through an active antioxidant mechanism in plants to counteract the damage caused by ROS production (Lokhande et al., 2020). The antioxidants work by searching, neutralizing, and removing the reactive species (Hasanuzzaman et al., 2020b). An assessment of biochemical responses in alfalfa plants showed high peroxidase (POD) and glutathione *S*-transferase (GST) activity upon its exposure to Ni. The data on antioxidant enzymes was also in corroboration with the expression transcripts of the *Prx1C* gene, showing elevated biochemical activity in plants as a function of plant defense (Helaoui et al., 2020).

Furthermore, carbon dioxide (CO_2_) fixation and assimilation activities are also greatly affected by metal toxicity. For instance, inhibition of CO_2_ assimilation was reported in wheat by excessive concentrations of Cd and Zn (Paunov et al., 2018). Rubisco activity was observed to be reduced due to toxic metals/metalloids stress, as they react with the thiol group of the enzyme and render denaturing it (Son et al., 2014). Nitrogen metabolism is a crucial mechanism in plant growth and development that suffers from metal stress. For example, Cr lowered nitrate and ammonia assimilation enzymes in *Cyamopsis tetragonoloba* by enhancing their destruction through protease increase (Sangwan et al., 2014).

### Molecular Responses

As a defense mechanism to toxic metals/metalloids stimuli, root growth is reduced and inhibited by terminating the mitotic activity (Campos et al., 2018). Pavlova (2017) detected a decrease in meristem mitosis in *Plantago lanceolate* due to Ni toxicity, inhibiting root elongation. Similarly, Cr has also been described to inhibit mitotic cell division by delaying and extending the cell cycle in rice (Sundaramoorthy et al., 2010). ROS production in response to toxic metals/metalloids stress can cause damage to cellular membranes, nucleic acids, lipids, and proteins (Hasanuzzaman et al., 2020b). For example, Cu and Zn caused severe damage to cellular structures in the Populus species (Benyó et al., 2016). Plants also produce various metal binding structures, chelating agents or ligands as a defense or detoxification mechanism, such as organic acids, amino acids, phytochelatins (PCs), and metallothionein (MT), among others (Raza et al., 2020, 2021a).

Although plants have different mechanisms to prevent metal ions from reaching the nucleus, the entry of metals/metalloids causes cross-linking of DNA and proteins, DNA mutation (deletion, addition, modification), alter the base structure of the DNA, or cause DNA strands to break (Emamverdian et al., 2015). They further disrupt lipids, damage Chl, disturb cell homeostasis, and also interfere with the electron transport chain and energy production and assimilation through ATP molecules, leading to programmed cell death (Parmar et al., 2013).

Exposure to Cd was found to display an enhanced expression of *Chit5* (one of the chitinase encoding genes) in roots by 7. 3-, 3. 9-, and 3.7-fold, compared with its expression in shoots in *Amaranth. cruentus*, *A. hypochondriacus* × *A. hybridus*, and *A. hypochondriacus* × *A. hybridus*, respectively (Lancíková et al., 2020). Alfalfa plant experienced a 25- and 29-fold increase in detoxifying enzyme superoxide dismutase (SOD) transcripts in response to bulk Cu and nano Cu, respectively (Cota-Ruiz et al., 2020). Research has established melatonin expression to help alleviate Cd stress in radish plants (Xu L. et al., 2020). Gao et al. (2020) demonstrated *PtrMTP* (*Populus trichocarpa* metal tolerance proteins) gene to play a significant role in inducing tolerance toward metal contamination in roots, stems, and leaves of *Populus trichocarpa* through homeostasis and detoxification mechanisms.

MicroRNAs have been reported to play a significant role in plant response and tolerance to toxic metals/metalloids stress by regulating chelation, antioxidant response, auxin signaling, and cytokinin signaling, among others (Ding et al., 2020). For instance, Casarrubia et al. (2020) reported microRNA to play a significant role in combination with the mycorrhizal association for regulating the response of *Vaccinium myrtillus* against Cd contamination. MicroRNA expression in terms of biosynthesis/biogenesis of various secondary metabolites has been found to play a major role in enhancing Al and Cd tolerance in transgenic tobacco (Cedillo-Jimenez et al., 2020). Notably, the expression of different microRNAs, including miR156, miR162, miR164, miR166, miR172, miR398, and miR408, were found to regulate the expression of their respective target genes in *Carthamus tinctorius* as a result of Cd exposure (Kouhi et al., 2020).

## Seven-(Omics)-Based Approaches to Improve Toxic Metals/Metalloids Tolerance in Plants

**FIGURE 2:**
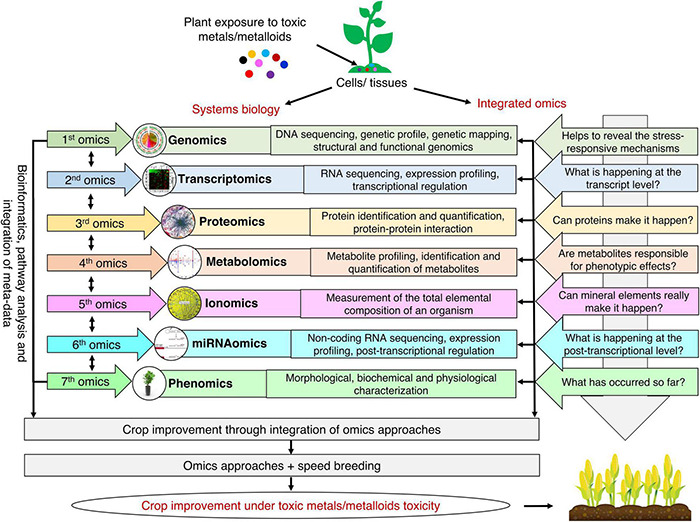
Integrated omics approach for developing toxic metals/metalloids tolerant plants. The use of multi-omics approach can help to reveal stress-responsive mechanisms at the genomic level, understand what is happening at the transcript and proteome level, provide clues about the interaction of metabolites with the phenotype, understand the role of different mineral elements, and unravel phenotypic changes in plants in response to toxic metals/metalloids toxicity. Integrating state-of-the-art omics approaches with speed breeding will help to meet the challenge of feeding a burgeoning human population.

Plant responses to toxic metals/metalloids toxicity rely on the regulation of molecular factors. Therefore, an integrated omics approach has been extensively used to comprehend the plant’s biological interactions and molecular mechanisms against toxic metals/metalloids toxicity. Regardless of the incredible advancements in genomics, it is essential to evaluate other omics tools for wide-ranging knowledge at the molecular level (Figure 2). Scientific investigations and existing information derived by omics tools target signaling pathways, key molecular regulators, and integrated mechanisms to enhance tolerance toward toxic metals/metalloids toxicity for crop improvement.

### Genomics

Genomics includes characterization, data sequencing, structural organization, genetic alignments, interactions, and functions of a whole organism (plant) (Gilliham et al., 2017). Genomics is interrelated to the terms like transcriptomics, metabolomics, transgenomics, epigenomics, and phenomics. These approaches, associated with high throughput (HTP) technologies, have made significant advancements in plant genomics research and have enabled the improvement of multiple important crop plants (Varshney et al., 2020, 2021a). Genomics can help to identify genes, enzymes, or other molecular factors involved in stimulating toxic metals/metalloids stress. Genomics-based findings and online genomic data sets provide a way forward to open new windows for multi-omics technologies and genome editing tools.

#### Quantitative Trait Locus Mapping

Various trait mapping methods, such as quantitative trait locus (QTL) mapping, QTL-sequencing (QTL-seq), and RNA-sequencing (RNA-seq), have been established to switch the fine-mapping course as it can distinguish candidate genes within major QTLs quickly. For example, the genetic architecture and regulation of Cd tolerance in barley plants identified two QTLs, one minor-effect and other major-effect QTL, with a phenotypic variance of 47.24 and 38.59%, respectively, associated with Cd tolerance. This analysis also identified 16 candidate genes linked with Cd tolerance (Derakhshani et al., 2020). Marker-trait association study was conducted for As related traits using 704 SNPs. About 9 QTLs were identified with a phenotypic variance ranged from 8.6 to 12.6%, out of which six QTLs (*qAsS2, qAsS5.1, qAsS5.2, qAsS6, qAsS9.1*, and *qAsS9.2*) for As content in shoots were mapped on chromosome 2, 5, 6, and 9, two QTLs in roots (*qAsR8.1* and *qAsR8.2*) on chromosome 8 and one QTL (*qRChlo1*) for relative Chl content was mapped on chromosome 1. Using these QTL intervals, 25 associated candidate genes were identified, showing transcription regulation for As toxicity-related traits (Murugaiyan et al., 2019). A total of four QTLs (*qAsTSL8, qAsTSL12, qAsTRL8*, and *qAsTRSB8*) for As phytotoxicity tolerance were found on chromosomes 8 and 12 using a composite interval mapping approach. A significantly effective QTL for As phytotoxicity tolerance in root contributing 24.9% phenotypic discrepancies was found on chromosome 8 with apparent pleiotropic effect on root-shoot biomass and shoot length (Syed et al., 2016).

QTL mapping in bread wheat for Al stress response revealed 79 QTLs, out of which 22 were putative, showing a range of phenotypic variance from 4.38 to 12.24%. Identified stable QTLs related to days to heading and grain yield were co-located with those of Al concentration with zero × additive environment interaction (Farokhzadeh et al., 2020b). Two bi-parental populations were used for QTL identification against Al toxicity in rice accessions. Fourty eight regions with a phenotypic variation of 57% were identified for Al tolerance, out of which four were co-localized with previously reported QTLs and three new regions (*ART1*, *STAR2*, *Nrat1*) on chromosome 1, 9, and 12 found inducing Al sensitive rice mutants. The study revealed that mapping with *Indica/Japonica* background identified QTLs where *indica* parent enhanced Al tolerance in *Japonica* background (Famoso et al., 2011). In another QTL map-based study of rice against Al resistance, a QTL named *Alt12.1*, was identified for Al resistance and ultimately considered *ART1* as a primary candidate gene for this QTL region. The presence of the *ART1* allele in different parental backgrounds has reportedly affected the expression of many genes in rice against Al tolerance (Arbelaez et al., 2017). A recent study in tomato identified 103 QTLs, out of which six QTLs were for fruit and leaf Fe contents while eight QTLs were for yield Fe content (FeUEc). Also, two relevant candidate genes encoding for specific proteins of tomato xylem sap were identified under Fe deficiency, thus affecting fruit yield and quality traits (Asins et al., 2020). Eight QTLs were detected for Al resistance in common bean (*Phaseolus vulgaris*) with a phenotypic variation of 7.6–14.7%. QTLs found were related to traits such as root length (RL), root dry weight (RDW), and shoot dry weight (SDW). This study identified the resistant Al activated transporter candidate gene (Phvul.007G025900) underlying the target QTL (Njobvu et al., 2020). QTL mapping for toxic metals/metalloids stress tolerance in rice identified one QTL related to Cu and Hg, three QTLs for As, and two QTLs for Fe and Zn contents. Candidate genes underlying the target QTLs for Zn (*LOC_Os01g14440, LOC_Os01g18584, LOC_Os01g20160*) and Fe (*LOC_Os04g34600*) were predicted to improve Zn and Fe stress tolerance in rice (Zhu et al., 2020). A total of 40 QTLs for Al stress tolerance were mapped on the A genome in wheat using composite interval mapping (CIM) and mixed composite interval mapping (MCIM) algorithms, which showed significant QTL × environment interactions (Farokhzadeh et al., 2020c). Another study in wheat revealed 58 QTLs associated with Al stress tolerance affecting spikelet-related traits (Farokhzadeh et al., 2020a). A further list of QTLs mapped for toxic metals/metalloids tolerance in different plant species is provided in Table 1.

**TABLE 1:** Summary of QTL/gene mapping for toxic metals/metalloids tolerance in different plant species.

Metals/metalloids	Plant species	QTLs/genes mapped	Number of lines/accessions used	Chromosome	Key observations	References
Cadmium	*Hordeum vulgare*	One minor and one major	87 DH lines	2H, 6H	One major-effect and one minor-effect QTL along with 16 candidate genes for Cd tolerance were detected.	Derakhshani et al., 2020
Aluminum	*Triticum aestivum*	79	RIL population (167 lines)	1, 4	79 QTLs were identified, some of which were stable and were associated with grain yield traits.	Farokhzadeh et al., 2020b
Aluminum	*Phaseolus vulgaris*	8	RIL population (150 lines)	Pv02, Pv04, Pv06, Pv07, Pv09, Pv1	Eight QTLs identified for Al resistance with a phenotypic variation of 7.6–14.7%. QTLs found were related to root length, root dry weight, and root fresh weight.	Njobvu et al., 2020
Aluminum	*Triticum aestivum*	40	RIL population (167 lines)	1A, 1B, 1D-a, 2A-b, 2A-d, 2B, 2D, 4A, 4B, 6A-a, 6B, 7A, and 7D	Nine out of 40 QTLs were putative detected by CIM method.	Farokhzadeh et al., 2020c
					20 additive and six pairs of epistatic stable QTLs identified by MCIM method.	
Iron	*Solanum lycopersicum*	14	RIL population (121 lines)	9, 12	Six QTLs identified for fruit and leaf Zn content, while eight QTLs identified for FeUEc.	Asins et al., 2020
					Two putative candidate genes were identified under Fe deficiency.	
Iron, zinc, copper, mercury, and arsenic	*Oryza sativa*	9	RIL population (120 lines)	1, 2	One QTL related to Cu, Hg; three QTLs for As; two QTLs for Fe and Zn contents were identified against metal ion stress.	Zhu et al., 2020

*DH, double haploid; RIL, recombinant inbred line; QTL, quantitative trait locus; CIM, composite interval mapping; MCIM, mixed composite interval mapping.*

#### Genome-Wide Association Study

Genome-wide association study (GWAS) is a method utilized in genetics to relate definite genetic variations with certain traits in different individuals. It overcomes many constraints of traditional trait mapping (QTL) by offering advanced resolution, often to the gene level, and using trials from formerly examined populations in which frequently occurring genetic variations can be coupled with a phenotypic difference. For example, a GWA study for low Cd accumulation in rice identified *OsABCB24* gene underlying a novel QTL (*qCd1-3*) (Pan et al., 2020). A recent GWA study in wheat identified five loci (*qSCd−1A*, *qSCd−1D*, *qZn−2B1*, *qZn−2B2*, and *qFe−6D)* associated with Cd stress tolerance (Safdar et al., 2020a). Analysis of a large collection of rapeseed accessions identified four QTLs and underlying candidate genes, including *GSTUs, BCATs, UBP13, TBR*, and *HIPP01*, responsible for Pb tolerance (Zhang et al., 2020a). GWAS investigation on rice grains for Fe and Zn traces have identified novel marker-trait associations with a phenotypic variation of 2.1–53%, which could be helpful to identify candidate genes for improving Fe and Zn tolerance (Bollinedi et al., 2020). Rice crop has also been studied for Fe toxicity using a GWAS approach, and three linkage disequilibrium (LD) blocks were found to mainly contribute to Fe omission. These LD blocks were detected on chromosomes 1, 2, 3, 4, and 7 for vegetative and generative stages, facilitating tolerance against Fe toxicity (Utami et al., 2020). Integration of approaches like genome-wide association, genome-wide epistasis (GWE), and gene expression proved to be an effective strategy for identifying novel QTLs related to Fe tolerance (69 genomic regions) across 19 chromosomes (Assefa et al., 2020). GWA study in barley for potassium (K) tolerance identified three primary QTLs responsible for K translocation and also identified some candidate genes for improving potassium-use efficiency (Ye et al., 2020).

Furthermore, a GWA study in bread wheat identified 534 significant MTAs for K-related traits, which included 11 stable loci and 16 M-QTLs. This study also identified potential candidate genes involved in critical pathways associated with stress tolerance, nutrient uptake, and sugar metabolism, which possessed the potential to develop K stress tolerant wheat cultivars (Safdar et al., 2020b). Zhao Z. et al. (2018) performed a GWA study and found six SNPs associated with four non-redundant QTLs significantly related to As accumulation. The loci, localized from 25.71 to 25.77 Mb on chromosome 1, co-localized with already reported QTLs (*CAsA1/CAsS1*), and just one candidate gene *GRMZM2G130987* was discovered. The identified gene encodes a protein with P-P-bond hydrolysis-driven protein transmembrane transporter activity and contributes to As ion transport. Other SNPs located on chromosome 2 were found within the *BAsA2/XAsA2* QTL. Five candidate genes are present in this QTL, and the *GRMZM2G125495* candidate gene encodes a protein with extracellular glutamate-gated ion channel activity. This study provides a solid reason to further study the gene’s function in As accumulation (Zhao Z. et al., 2018). Liu X. et al. (2019) performed a GWA study against toxic metals in rice grain and found 22, 17, and 21 QTLs for grain associated with As, Cd, and Pb toxicity, respectively. The authors examined the candidate gene in *qGAS1*, a QTL for grain arsenic, with the best *P*-value found for the whole population. Toxin extrusion and transport protein of the multidrug might be the candidate gene for this QTL (Liu X. et al., 2019). Examples of GWA studies conducted in different crop plants for identifying toxic metals/metalloids tolerance loci have been described in Table 2.

**TABLE 2:** Summary of key GWA studies for toxic metals/metalloids toxicity in different crop plants.

Metals/metalloids	Plant species	Platform	No. of QTLs	No. of lines/accessions used	Chromosome	SNPs	Key observations	References
Cadmium	*Brassica napus*	Illumina Brassica SNP60 Bead chip	25	419	A3, A5, A9, C3, C5, C8	98	QTLs identified for root, shoot, and for Cd translocation. Homologs of key *Arabidopsis* genes identified that can be further used for Cd tolerance in other plants.	Chen et al., 2018
Cadmium	*Oryza sativa*	SLAF-seq, Illumina-HiSeq 2500	35	338	1, 2, 3, 4, 5, 6, 7, 8, 9, 11, 12	203	Identified 35 significant QTLs for low Cd accumulation, including a novel QTL, *qCd1-3.*	Pan et al., 2020
							Differential expression of *OsABCB24*, a candidate gene underlying *qCd1-3.*	
Cadmium, iron, and zinc	*Triticum aestivum*	Illumina iSelect 90K	5	120	1A, 1D, 2B, 6D	179	Five novel loci detected to be associated with Cd toxicity.	Safdar et al., 2020a
Copper	*Triticum aestivum*	Wheat 660K SNP assay	4	243	1D, 6A, 6B, 7D	489	Four significant QTLs with a phenotypic variation of 4.71–8.66% regulating GCC in wheat were observed.	Zhao et al., 2020
Lead	*Brassica napus*	60 K Brassica Infinium SNP array	4	472	A9, C3, C4	9	Identified four QTLs and nine candidate genes associated with Pb tolerance.	Zhang et al., 2020a
Iron and zinc	*Oryza sativa*	50 K SNP chip	29	192	1, 2, 3, 4, 6, 7, 8, 9, 10	31,132	Total of 29 marker-trait associations (MTAs) were identified, showing a phenotypic variation of up to 53% for traits controlling Fe and Zn contents.	Bollinedi et al., 2020
Iron	*Oryza sativa*	384 SNP chip	8	288	1, 2, 3, 4, 7	384	Three LD blocks containing QTLs for Fe toxicity tolerance were found that can be used for rice breeding programs for specific land types.	Utami et al., 2020
Iron	*Glycine max*	Illumina Infinium SoySNP50K BeadChip	69	460	3, 5, 16	36,000	Integration of approaches like genome-wide association (GWA), genome-wide epistasis (GWE), and gene expression enabled identification of novel Fe tolerance QTLs, with a significant QTL found on chromosome Gm03.	Assefa et al., 2020
Potassium	*Hordeum vulgare*	Diversity Array Technology (DArT)	3	179	1H, 6H	13,634	Identified three significant QTLs associated with K uptake and translocation.	Ye et al., 2020
Potassium	*Triticum aestivum*	90 K Infinium SNP array	11	150	1A, 1B, 1D, 2A, 3A, 3B-I, 3B-II, 4A-I, 4A-II, 4B, 5B-I, 5B-II, 6A, 6B, 7A, and 7B	20,853	Total of 534 significant MTAs were identified for potassium related traits, which included 11 stable loci and 16 M-QTLs.	Safdar et al., 2020b
							Identified potential candidate genes involved in crucial pathways related to stress tolerance, nutrient uptake, and sugar metabolism.	
Aluminum and iron	*Oryza sativa*	44 K SNP array	6	373	1, 2, 9, 12	36,901	Identified forty eight regions associated with Al tolerance. Six Al tolerant QTL were detected for root growth, out of which three (*ART1*, *STAR2*, *Nrat1*) were used to induce Al sensitive rice mutant.	Famoso et al., 2011
							Promoted the selectively introgressing alleles for trait enhancement	

#### CRISPR/Cas9 System

Clustered regularly interspaced short palindromic repeats (CRISPR)/CRISPR-associated protein 9 (Cas9) system is a valuable editing tool with high efficiency, specificity, and possessing a wide range of applications (Zafar et al., 2020). Targeted knock-in/out, deletion, insertion, and substitution mutations generated by CRISPR/Cas9 system have explored regulatory functions of genes and their impact on other biochemical processes and have helped to improve many crops by increasing their scavenging capacity under toxic metals/metalloids toxicity (Table 3).

**TABLE 3:** Summary of genome editing studies for toxic metals/metalloids tolerance in different plant species.

Metals/metalloids	Plant species	Gene target	Modification	Key observations	References
Iron	*Arabidopsis thaliana*	*GSNOR*	Knock-out	Mutants were found to be sensitive to high Fe toxicity, showing this gene to generate tolerance in roots against Fe stress.	Li et al., 2019
Cadmium	*Oryza sativa*	*OsABCG36*	Knock-out	Targeted gene could transport Cd out of the cell to detoxify its effect. Mutants were tolerant to Cd accumulation in roots, but not in shoots.	Fu et al., 2019
Cadmium	*Arabidopsis thaliana*	*AtPDF2.6*	Knock-out	Loss of *AtPDF2.6* gene reduced tolerance against Cd and was significantly accumulated when exposed to Cd stress. Mainly expressed in root tissues.	Luo et al., 2019
Cadmium and manganese	*Oryza sativa*	*OsNRAMP1*	Knock-out	Reduced uptake and transportation of Mg, Fe, Cd, and As.	Chang et al., 2020
Cadmium	*Solanum lycopersicum*	*class II glutaredoxin*	Knock-out	Knockout mutation on members of class II glutaredoxin (GRXs) against Cd toxicity protected chloroplasts of cells.	Kakeshpour, 2020
Zinc and copper	*Oryza sativa*	*OsZIP9*	Knock-out	Higher concentration of Zn improved the growth of plants.	Yang M. et al., 2020
Iron and zinc	*Oryza sativa*	*OsIRO3*	Knock-out	Accumulation of ROS. Maintenance of Fe homeostasis by tolerating Fe deficiency or toxicity.	Wang et al., 2020c
Zinc	*Arabidopsis thaliana*	*OZS3*	Knock-out	Complete loss of the *OZS3* gene resulted in reduced growth, early flowering and long petioles.	Weber et al., 2020

Knock-out of *OsNRAMP1* gene responsible for the uptake of metals like Cd, Fe, As, and Mn, significantly reduced the uptake of Cd and Mg and their storage in rice shoots and grains (Chang et al., 2020). Furthermore, *OsIRO3* gene knock-out using the CRISPR/Cas9 approach highlighted the function of this gene in the regulation of Fe homeostasis. *OsIRO3* gene mutants accumulated ROS, and their growth and development were affected under Fe deficiency (Wang et al., 2020c). Single, double, and triple mutants developed against Cd toxicity in tomato by CRISPR/Cas9 system were found to protect chloroplasts by mutating members of class II glutaredoxin (GRXs) from Cd toxicity (Kakeshpour, 2020). Knock-out mutant lines generated by CRISPR/Cas9 system in rice targeting *OsZIP9* gene against Zn toxicity showed a substantial reduction in growth, which revealed Zn as an essential element for growth (Yang M. et al., 2020). In *Arabidopsis, OZS3* gene knock-out reduced the growth of roots and elusive development of plants, further damaged embryos, and caused early flowering (Weber et al., 2020). Cd accumulation was studied in rice by knocking out a segment of the *OsABCG3* gene via CRISPR/Cas9 technology. Knock-out mutants accumulated Cd in roots cells and routed Cd contents out of the cell sap to detoxify its effect, thus generating tolerance against Cd (Fu et al., 2019). In *Arabidopsis*, the variant *GSNOR* has been observed to generate tolerance and promote root growth against Fe toxicity. The mutant lines produced using the CRISPR/Cas9 vector were also found to be highly sensitive toward high Fe content, which affected the whole seedlings and produced leaves that were smaller than the wild type (Li et al., 2019). Loss of *AtPDF2.6* gene has been reported to reduce tolerance against Cd along with its significant accumulation in root cells when exposed to Cd stress (Luo et al., 2019). Genome editing using the CRISPR/Cas system has a promising future for sustainable agricultural production to feed the world’s growing population. In the near future, more genome editing works need to be performed under the toxicity of different metals/metalloids like As, Pb, Hg, Ni, Cr, etc., in different crop plants to explore the stress resistance mechanisms.

### Transcriptomics

The term transcriptomics refers to a set of techniques that are used to study the RNA transcripts in an organism. Several transcriptomic techniques are widely used to measure the abundance of the transcripts of interest (Lowe et al., 2017; Mehmood et al., 2021; Raza et al., 2021c). Studies have shown that stress-induced changes in gene expression may lead to the synthesis of novel proteins, stress mediating metabolic compounds, or encode transcription factors (TFs), which help to regulate the stress-responsive genes (Table 4).

**TABLE 4:** Summary of key transcriptomics, proteomics, metabolomics, and ionomics studies under toxic metals/metalloids toxicity in different plant species.

**Transcriptomics**						
**Plant species**	**Stress conditions**	**Target tissues**	**Approach**	**Functional annotation methods**	**Key observations**	**References**
*Phytolacca americana*	50 mg kg^–1^ CdCl_2_; 15 days	Roots, leaves, and stem	RNA-Seq	NR, SWISS-PROT, GO, KEGG	1,515 differentially expressed genes (DEGs) were identified. 12 DEGs validated using qRT-PCR. Genes related to toxic metal tolerance identified including nicotianamine synthases (8), ABC transporter (3), expansins (11), metallothionein (3), ZRT/IRT protein (4), and aquaporins (4)	Chen et al., 2017
*Brassica juncea*	25 μM CdSO_4_; 24 h	Roots	Microarray	Gene chip Arabidopsis ATH1 genome array	38 DEGs identified, and six DEGs validated by qRT-PCR. The DEGs were mainly involved in Cd metabolism.	Dalyan et al., 2017
*Oryza sativa*	100 μML^–1^ AlCl_3_; 24 h	Root tips	RNA-seq	KEGG, WEGO 2.0	14,550 DEGs identified, of which most were related to Al tolerance. Total of 92 genes were reported to be linked with different pathways that mediated Al-induced inhibition in plants.	Zhang et al., 2019c
*Verbena bonariensis*	100 mg kg^–1^ CdCl_2_; 20 days	Root	RNA-seq	GO and KEGG	23,424 DEGs identified. 10 DEGs validated by qRT-PCR. DEGs encoding lignin synthase, chalcone synthase, and anthocyanidin synthase identified under Cd stress.	Wang et al., 2019
*Triticum aestivum*	100 μM CdCl_2_; 24 h	Roots	RNA-seq	GO-GO network and pathway network analysis	1,269 and 399 DEGs identified in low and high Cd accumulation genotypes. Six genes validated using qRT-PCR. DEGs related to Cd uptake and transport include antioxidant defense, ATP binding, plant hormone signal transduction, and phenylpropanoid biosynthesis.	Zhou et al., 2019
*Vicia faba*	5, 10, 15, 20, 25 μM U; 72 h	Roots	RNA-seq	NR, KOG, GO, Swiss-Prot, eggNOG, KEGG, Pfam	4,974 DEGs identified. The uranium induction significantly up- and down-regulated 1,654 and 3,320 genes, respectively, involved in the regulation of cell metabolism and other processes, and processing of environmental and genetic information.	Lai et al., 2020
*Fagopyrum tataricum*	2,000, 10,000 bmgkg^–1^ Pb(NO_3_)_2_; 72 h	Leaves	RNA-seq	GO, KEGG	12,595 DEGs identified. Majority of DEGs were associated with phenylpropanoid synthesis pathway and up-regulated the expression of MAPKs and GSH metabolic genes along with the regulation of plant protecting metabolites and hormones.	Wang et al., 2020d
*Medicago sativa*	50, 150, 250, 500 mg kg^–1^ NiCl_2_; 60 days	Roots and shoots	RNA-seq	Fern Base, NCBI	Highly expressed prx1C, GST, and PC genes in roots and shoots actively mediated the negative impact of Ni on plant growth.	Helaoui et al., 2020
*Oryza sativa*	15 mM FeSO_4_; 2 days	Roots and leaves	RNA-seq	Top GO, Ensembl Plants, TAIR	1,147 and 1,038 DEGs identified under control and Fe treatment. The Fe stress affected “Hacha” genotype more abundantly by causing alterations in roots’ gene expression pattern. Total of 1,248 and 1,161 DEGs were less abundant in “Lachit” roots under control and Fe stress conditions.	Kar et al., 2020
*Dendrobium officinale*	2, 5, 9, 14 mg L^–1^ CdSO_4_; 30 days	Roots	RNA-seq	GO and KEGG	2,469 DEGs identified. DEGs helped identify complex metabolic pathways and regulated the transcription factors involved in regulating Cd stress.	Jiang et al., 2020

**Proteomics**						

**Plant species**	**Stress conditions**	**Target tissue**	**Extraction protocol**	**Analytical approach**	**Key observations**	**References**

*Artemisia annua*	100 μM As + 100 μM Se; 3 days	Roots, shoots	TCA	2D- PAGE, MALDI-TOF-MS	20 differentially abundant proteins (DAPs) identified. The DAPs were involved in energy metabolism, secondary metabolism, photosynthesis, transcriptional regulators, transport proteins, and lipid metabolism.	Kumari and Pandey-Rai, 2018
*Capsicum annuum*	0 or 100 ppm Na_2_SeO_4_; 24 h	Shoots	EDTA	LC-MS/MS	4,693 DAPs identified. Identified DAPs were associated with protein processing, post-translational modification, chaperones, protein turnover, and metabolic process.	Zhang et al., 2019a

**Proteomics**						

**Plant species**	**Stress conditions**	**Target tissue**	**Extraction protocol**	**Analytical approach**	**Key observations**	**References**

*Eucalyptus camaldulensis*	30, 50, 100 μM, CuSO_4_⋅5H_2_O; 6 weeks	Leaves, roots	TCA/acetone	MS	26 DAPs were identified. 11 DEPs were up-regulated, and 15 DAPs were down-regulated. Identified DEPs were involved in antioxidant enzymes, photosynthesis, metabolism, transcription, and translation.	Alotaibi et al., 2019
*Arachis hypogaea*	2 μM CdCl_2_; 7 days	Roots	TCA/Acetone	LC-ESI-MS/MS, RT-PCR	30 DAPs were found to be linked with heavy metal transport, while 86 DAPs were found to be associated with cell wall modification.	Yu R. et al., 2019
*Stylosanthes guianensis*	5 or 400 μM MnSO_4_; 10 days	Shoots, roots	Tris-HCl	LC-MS/MS	356 DAPs identified. 172 DAPs were strongly induced, while 96 DAPs were completely suppressed. Identified DAPs were involved in carbon fixation, defense response, signaling, metabolism, photosynthesis, and cell wall modulation.	Liu P. et al., 2019
*Oryza sativa*	25 μM AsIII, NaAsO_2_ + 25 μM SeIV, Na_2_SeO; 15 days	Roots, shoots	Acetone	MALDI-TOF/TOF, qRT-PCR	Significantly enhanced expression of 14,303 proteins for As + Se exposure, compared to As alone. In As stress, Se application effectively mitigated As toxicity, improving plant growth via regulation of 14-3-3 proteins. FBPase, AtpB, GLN1, and GLN2 proteins were found to be involved in defense, photosynthesis, and energy metabolism upon Se exposure.	Chauhan et al., 2020
*Setaria italica*	120 g hm^–2^ Na_2_SeO_3_; 72 h	Grains	HEPES-based buffer	LC-MS/MS	123 DAPs identified. The DAPs were mainly involved in amino acid and carbohydrate metabolism.	Liang et al., 2020
*Nicotiana tabacum*	5.36 mg kg^–1^ Zn^+2^; 10 days	Leaves	TCA/Acetone	LC-MS/MS	Zn stress resulted in the down-regulation of 8 proteins. Chl synthesis was not inhibited significantly, and only a few proteins involved in the electron transport chain showed down-regulation. Zn-stress did not significantly inhibit photosynthetic function in tobacco leaves.	Zhang et al., 2020b
*Allium cepa*	5–15 μM Pb(NO_3_)_2_; 6, 12, and 24 h	Roots	Tris-HCl	2-DE, AutoFlex TOF/TOF II-MS	17 DAPs identified. Lowered expression of *Anx D1*, *SHMTI*, and *COMT2* resulted in decreased defensive response, respiration, and the response of other functions, respectively. Improved expression of *NDPK*, *PR1*, and *CHI1* resulted in increased transcription, translation, and better pathogen invasion, respectively.	Lyu et al., 2020
*Cichorium intybus*	100, 200, and 300 μM Pb; 46 days	Leaves	Tris-HCl	SDS-PAGE	81 DAPs identified. Total of 16 proteins were up-regulated and 13 were down-regulated. Identified proteins were associated with plant-stress response and adaptation toward metal toxicity.	Malik and Pirzadah, 2020

**Metabolomics**						

**Plant species**	**Stress conditions**	**Target tissue**	**Analytical platform**	**Data analysis**	**Key observations**	**References**

*Cucumis sativus*	10, 100, and 500 mg L^–1^ CuSO4; 7 days	Leaves	GC-TOF-MS, LC-MS/MS	PLS-DA	Total of 149 primary and 79 secondary metabolites were quantified. 1.4–2.4-folds of intermediates involved in TCA were found to be down-regulated upsetting carbohydrate metabolism.	Zhao L. et al., 2018
*Glycine max*	0.1–100 mg L^–1^ Mo; 48 h	Leaves, roots	UPLC, LC-MS	PCA, OPLS-DA, KEGG	Identified 42 and 19 significantly different metabolites (SDMs) in roots and leaves, respectively. Organic acids, gluconic acid, D-glucarate, and citric acid were amplified by 107. 63-, 4.42- and 2.87-folds after Mo exposure. Organic compounds such as 2-oxoarginine, L-nicotine, gluconic acid, D-glucarate, and citric acid played a significant role in chelating Mo and decreasing its toxicity.	Xu et al., 2018
*Oryza sativa*	400 ppm FeSO_4_.7H_2_O; 10 days	Roots, shoots	GC-MS	PCA, PLS-DA	Levels of elaidic acid increased, while linoleic- and linolenic acid decreased. In shoot and root, alteration of the fatty acid composition suggested metabolites alteration.	Turhadi et al., 2019

**Metabolomics**						

**Plant species**	**Stress conditions**	**Target tissue**	**Analytical platform**	**Data analysis**	**Key observations**	**References**

*Glycine max*	25 μM Fe (III)−EDTA; 10 days	Roots, leaves	GC-MS	OPLS-DA	N assimilation was inhibited, which reduced proteins in roots and nodules. Sugars increased or maintained at a constant level in different tissues under Fe deficiency, which probably relates to oxidative stress, cell wall damage, and feedback regulation. Increased levels of ascorbate, nicotinate, raffinose, galactinol, and proline in different tissues possibly helped resist the oxidative stress induced by Fe deficiency.	Chu et al., 2019
*Helianthus annuus*	1, 5, and 25 mg L^–1^ Cr(VI); 7 days	Roots	capHPLC-ESI-QTOF-MS	PLS	70% of metabolites involved in LA metabolic pathway are affected by Cr(VI) stress. Detection of four EKODE isomers not included in LA metabolism and found only in the exposed roots. Oxidation of LA to HpODE isomers upon incubation with Cr(VI).	Ibarra et al., 2019
*Brassica napus*	100 μM CdCl_2_.2.5H_2_O; 8 days	Leaves	UPLC/MS	PCA, PLS-DA, KEGG	644 SDMs found in sensitive genotype ZD622, and 487 SDMs in tolerant genotype CB671. Most SDMs were involved in Cd-mediated stress tolerance pathways.	Mwamba et al., 2020
*Elodea nuttallii*	280 μg L^–1^ Cd as Cd(NO_3_)_2_; 24 h	Shoots	GC-MS, LC analysis	PCA, MetaboAnalyst KEGG	Cd stress caused significant variations in aminoacyl-tRNA biosynthesis and branched-chain amino acid pathways. In the shoot, Cd induces a concentration of 11 amino acids, 2 sugars, adonitol, and pipecolic acid in the cytosol, and Cd induces a concentration of glycine, ammonium, hydroxy.	Cosio and Renault, 2020
*Cucumis melo* L.	300 μmol L^–1^ CuSO_4_; 3 days	Roots	UPLC/MS	KEGG	70 DEGs identified; 42-downregulated and 28-upregulated. 318 SDMs identified, 150-downregulated and 168-upregulated. Identified SDMs and DEGs were involved in JA biosynthesis; comprising lipoxygenase related genes, and lecithin and linoleic acid metabolites.	Hu et al., 2020
*Cucumis sativus* L.	3 μM Se (Na_2_SeO_3_)–50 μM Cd (CdCl_2_); 7 days	Leaves, roots	GC-MS	OPLS-DA, PCA, HCA, KEGG	Intermediates of TCA, glycolysis, and some amino acids were upregulated. Differentially regulated metabolites have a significant role in developing Se-mediated Cd tolerance.	Sun et al., 2020
*Vicia faba*	25 μM U [UO_2_(NO_3_)_2_⋅6H_2_O, 238U]; 72 h	Roots	GC-MS	KEGG	53 SDMs identified to be related to carbohydrate metabolism; including 12-downregulated and 13-upregulated metabolites. U led to the imbalance of the expression of related metabolites in the energy metabolism pathway of plant cells.	Zhang et al., 2020d
*Ipomoea batatas L.*	1.68–5.16 mg kg^–1^ U, 0.78–2.02 mg kg^–1^ Cd; 150 days	Roots	UPLC-MS	PCA, OPLS-DA, KEGG	634 SDMs identified in U + Cd; including 428 up-regulated and 214 down-regulated metabolites. Induced expression of plant hormones and cyclic nucleotides in cells. Regulated primary and secondary root-metabolism to induce U and Cd toxicity resistance.	Zhang-Xuan et al., 2020

**Ionomics**						

**Plant species**	**Approach**	**Element**		**Tissue**	**Key observations**	**References**

*Zea mays*	ICP−OES	Cd, Mo, Ca, Cu, Fe, K, Mg, Mn, P, S, and Zn		Shoot	Significant genotypic variation found among all minerals. *ZmHMA2/3* and *ZmMOT1* were found to be responsible for Cd and Mo contents in shoot.	Stich et al., 2020
*Brassica napus*	ICP−OES	B, Ca, Cu, Fe, K, Mg, Mn, Na, P, S, and Zn		Shoot and root	Total of 133 and 123 QTLs identified for the shoot and root ionome under OP and LP. Six QTL clusters were identified to be influencing mineral elements.	Wang et al., 2020e
*Oryza sativa*	Ex-3600 ED-XRF spectrometer	F, Co, Si, Ca, K, S, Zn, Cu, Ni, Fe, Mn, V, and Se		Seedling	Reduced fluoride toxicity and stimulated plant growth.	Banerjee et al., 2020
*Solanum tuberosum*	ICP-AES	Co, Zn, Cd, and Pb		Tubers	Reduced contamination of heavy metals in potato tubers	Muntean et al., 2019
*Oryza sativa*	ICP-MS	As, B, Ca, Cd, Cu, K, Mg, Mn, Mo, Na, Ni, P, Zn, and Ti		Straw and grain	Identified 70 novel ionomic QTLs and *OsMOT1* as a causative gene underlying a QTL controlling Mo tolerance.	Wang et al., 2020a

Transcriptomic techniques represent an effective method to enhance toxic metals/metalloids tolerance in plants by better understanding signaling mechanisms and gene ontology (GO). The transcriptomic profile screening of two contrasting varieties of rice (Fe susceptible “Hatcha” and Fe tolerant “Lachit”) revealed 22 out of 35 metal homeostasis genes in the tolerant variety. Furthermore, leaf transcriptome showed a more pronounced response in Hatcha, leading to a high degree of differential gene regulation (Kar et al., 2020). High and low grain Zn and Fe containing wheat genotypes revealed enrichment of GO terms such as Zn and Fe binding, Chl synthesis, ATP-synthase coupled transport, and oxidoreductase activity (Mishra V. K. et al., 2019). The molecular insights into transcriptome expression profiling of chickpea under toxic metals/metalloids [As(III), Cr(III), and Cd(II)] showed induction of crucial metabolic pathways under stressed conditions. Moreover, nine genes that played a major role in regulating these metabolic pathways were found to be differentially expressed in response to stress conditions (Yadav et al., 2019). Recently, Di et al. (2021) studied the stress-responsive genes of upland rice exposed to As(III) and As(V). Under metals toxicity, many genes were down-regulated compared to those that were up-regulated. Arsenic treatment resulted in unique transcriptome profiles changes, and a novel set of typical response genes were found. The antioxidant enzyme activities were consistent with the antioxidant enzyme-related genes expression, and several transports and defense enzyme-related pathways were identified (Di et al., 2021). Shukla T. et al. (2018) studied the response of *Arabidopsis* accessions under As(V) toxicity. Differential transcriptome modulation was found in sensitive “Slavi-1” and tolerant “Col-0” accessions. The results showed that As-induced genes are linked with stress response and detoxification pathways (Shukla T. et al., 2018).

According to Komarkova et al. (2020), Cd resulted in alteration of poplar’s gene transcripts, which not only regulated the plant defense mechanism through differential gene expression but also led to the production of phytohormones. Additionally, long-term exposure to stress was found to reduce the Cd toxicity in plants (Komarkova et al., 2020). Another transcriptomic study carried out on Chinese flowering cabbage (*Brassica parachinensis*) showed that Cd toxicity was mediated through some important tolerance-inducing genes, including *HMA3, HMA4*, and *Nramp1* (Wang et al., 2017). Furthermore, the genetic insights in two contrasting wheat genotypes (low Cd accumulating L17 and high Cd accumulating H17) revealed 1,269 genes to be differentially expressed. These genes showed heme-binding as the most active GO network, followed by metal binding. In contrast, phenylpropanoid biosynthesis and glutathione metabolism were found to be the major pathways active under Cd stress (Zhou et al., 2019). The analysis of the transcriptional data set of *Verbena bonariensis* under Cd stress revealed ROS scavenging system, photosynthesis, transpiration mechanism, chelating reaction, and production of secondary metabolites associated with DEGs as analyzed through GO and KEGG pathways (Wang et al., 2019).

The transcriptional analysis of the creeping bentgrass identified four transcription factors (bZIP, WRKY, MYB, and ERF) linked with Cd stress (Yuan et al., 2018). In a Cd-hyperaccumulator, *Siegesbeckia orientalis*, the comparative transcriptome analysis of roots with and without Cd treatment revealed a high number of DEGs, indicating the involvement of multiple biological pathways to cope up with Cd stress at the molecular level (Xu X. et al., 2020). Among such functional pathways, the ubiquitin-proteasome system (UPS) in plants has been reported to act through sequential actions of a cascade of enzymes. A comparative transcriptional study on a Cd hyperaccumulator *Viola baoshanensis* and its non-tolerant counterpart *Viola inconspicua* showed overexpression of genes involved in the UPS pathway under Cd exposure in *V. baoshanensis*, thereby supporting the hypothesis that high transcript levels of the genes involved in the UPS pathway can enhance tolerance to Cd toxicity (Shu et al., 2019).

Another study on the transcriptomic response of alfalfa plants, while assessing its phytoremediation potential for Ni, showed high expression of peroxiredoxin-1C, glutathione-S-transferase (GST), and phytochelatins (PCs). These genes were linked to an antioxidative response, prevention of cell damage, and Ni detoxification through its binding with PCs forming Ni-PC complexes, respectively (Helaoui et al., 2020). Kök et al. (2020) evaluated B accumulator *Puccinellia distans* for Se tolerance. Here, RNA-seq data showed induction of Se assimilation and stress response genes under stress conditions, which in turn altered the expression of gene transcripts involved in developmental (2.2%), transcriptional and translational (7.3%) processes, biotic (2.2%), and abiotic (17.7%) stress responses in *P. distans.* It further restricted the movement of Se by trapping it in the cell wall through the up-regulation of lignin production-related transcripts (Kök et al., 2020). Transcriptomic analysis of the Co stress response in *Salix babylonica* showed 2,002 DEGs, out of which 1,165 were identified in root and 837 in shoots. In addition, 107 transcription factors were identified from the DEGs, and most of them were reported to belong to the NAC and ERC families (Wang et al., 2020f). Another technique, translating ribosome affinity purification (TRAP) has been used to study transcriptional program in *Arabidopsis* against Fe stress. Using this technique, the excess Fe was sensed in *opt3* leaves. This study highlighted the first tissue, vasculature, in comparison with roots and leaves to respond against Fe deprivation and supply, thus maintaining Fe homeostasis through xylem and phloem. Moreover, a total of 1,143 DEGs were identified with more than 2-fold change, including 539 DEGs in roots and 604 DEGs in leaves, thus an up-regulation of iron deficiency responsive genes (Khan et al., 2018).

Laf vasculature from *Arabidopsis* leaves have also been used to execute single-cell RNA analysis and identified 19 clusters of all cell types, including mesophyll, guard cells, vascular cells, hydathodes, and some metabolic pathways to recognize their roles. The results identified potential roles of these clusters in sugar transport, amino acid transport, hormone biosynthesis, and defense-related responses (Kim et al., 2021). Single-cell transcriptome analysis has not been studied so far. Yet, there is a dire need to implement single-cell transcriptome analysis against the toxic metals/metalloids toxicity and may provide key resources and molecular insights to develop new strategies regulating the flux of ions, signals, and metabolites. Taken together, the analysis of transcriptomics data obtained through various studies suggests that it not only brings useful insights to functional genomics but also has a great potential to support molecular breeding, genetic engineering, phytoremediation, and metal complexation pathways. Meanwhile, there is a need to make smart use of comprehensive tools to integrate transcriptomics with other omics data and get useful insights into unidentified linkages. Summary of some experiments performed using transcriptomic approaches under toxic metals/metalloids toxicity is discussed in Table 4.

### Proteomics

Proteomics comprehensively covers the encoded proteins in living organisms at a particular instance and plays a vital role in understanding all cellular routes at the molecular level (Mehmood et al., 2021). Recently, proteomics has appeared as a vital tool to convey data on the survival of plants and adaptation toward toxic metals/metalloids toxicity in several plants (Table 4). From a methodological viewpoint, proteomics technology has progressed quite fast from the first generation (i.e., two-dimensional electrophoresis-mass spectrometry (2DE-MS); to second-generation (i.e., isobaric/isotopic tagging); to third-generation (i.e., shotgun and gel/label-free approaches); and lastly to fourth generation (i.e., mass western, targeted, SRM/MRM approaches) (Jorrin-Novo et al., 2019). Innovative proteomic systems provide a comprehensive understanding of metal-responsive proteins for plant stress tolerance (Table 4).

For instance, plant vascular systems like xylem sap transport minerals, water, and toxic metals (Cd) from roots to shoots. Proteomic variations in xylem sap significantly detoxify Cd in plants (Luo and Zhang, 2019). *Brassica* seedlings were treated with Cd (0 and 10 μM) for 3 days, and the collected xylem sap was subjected to lyophilization. Notably, 672 proteins from the xylem sap of Cd-treated rapeseed plants were identified through proteomic analysis (LC-MS/MS, shotgun). Most affected metabolic pathways between these proteins were found to be linked with stress/oxidoreductases, protein/lipid metabolism, and cell wall modification. Protein-like defensins, *BnPDFL*, found in xylem sap behaved as a Cd-chelating agent, confirming its positive role in regulating Cd tolerance (Luo and Zhang, 2019). In another study, the iTRAQ approach illustrated dynamic changes in root proteome in maize seedlings (Wen et al., 2019). Plant’s roots were exposed to 200 mg L^–1^ CdCl_2_, and both root and shoot growth were found to be severely inhibited in the first 72 h. The different numbers of differentially abundant proteins (DAPs; 733, 307, 499, and 576) were isolated after 12, 24, 48, and 72 h, respectively. These DAPs displayed numerous functions like energy and carbohydrate metabolism, ribosomal synthesis, cellular metabolism, ROS homeostasis, and cell wall organization. Amongst these GSTs, *GRMZM2G308687* showed extra abundance after 12, 48, and 48 h Cd-treatment. GST protein was found to be mainly tangled in PCs generation for Cd tolerance (Wen et al., 2019). Label-free proteomic analysis of Cd treated *Iris lactea* revealed 163 and 196 DAPs expressed in shoots and roots, respectively. Bioinformatic studies revealed that these DAPs, which are responsive to Cd, were majorly involved in redox reactions, signal transduction, ion transport, and other biochemical mechanisms. They were not only involved in lignin and amino acid biosynthesis pathways but also assisted GSH and glycerolipid metabolism. From *I. lactea*, a mannose-specific lectin (Cd-induced) was found to increase Cd-sensitivity and enhance Cd-accumulation in yeast (Liu et al., 2020).

Chauhan et al. (2020) performed a transcriptomic and proteomic analysis in rice to explore the molecular cross-talk involving Se-mediated tolerance of As-toxicity. Se supplementation restored the structural deformities caused by As, which comprised the cell wall and membrane disintegration. As-transporter gene expression viz., *ABCG5, NIP1;1, NIP2;1, TIP2;2, NRAMP1, NRAMP5*, and sulfate transporters *SULTR3;1, SULTR3;6* were found to be high in As + Se treated plants when compared to As alone. This not only resulted in low As concentration but also reduced the toxicity. During As + Se exposure, the GST, GRX, and PRX up-regulation also confirmed that elevation of As resulted in oxidative stress (Chauhan et al., 2020). Proteomic analysis (LC-MS/MS) of Se-treated pepper seedlings revealed up-regulation of 172 proteins, while 28 proteins were found to be down-regulated. Identified DAPs were mainly associated with metabolic processes, protein turnover, protein processing, post-translational modifications, and chaperones. Furthermore, various heat shock proteins (HSPs) were also identified as DAPs, which helped to cope with metal toxicity (Zhang et al., 2019a). Zeng et al. (2019) performed quantitative proteomics (iTRAQ) to study differential protein expression in Se-enriched and non-Se-enriched rice seedlings. Overall, 3235 proteins were detected, of which 3,161 proteins were quantified from 401 DAPs. Interestingly, 77 targeted significant DAPs were screened further and classified into 10 sets comprising of actin, synthetases, hydrolases, tubulin, ligases, lyases, isomerases, heat shock proteins, oxidoreductases, and transferases. These findings indicated that active oxygen metabolism, anti-stress, anti-oxidation, amino acid, and carbohydrate metabolism of Se-enriched rice seedlings was higher as compared to non-Se-enriched plants (Zeng et al., 2019).

In another study, proteomic analysis of Cu-treated young seedlings of *Eucalyptus camaldulensis* revealed 26 targets taking part in protein expression. Elevated Cu levels up-regulated the expression of 11 proteins and down-regulated expression of 15 proteins. Identified proteins were associated with antioxidant enzymes, photosynthesis, metabolism, transcription, and translation (Alotaibi et al., 2019). Furthermore, Ceballos-Laita et al. (2018) used two different proteomic approaches, i.e., shotgun and 2-DE, to study the effects of Mn toxicity on tomato root proteome. The shotgun approach identified 367 reliable proteins, while 2-DE yielded 340 consistent spots. A total of 54 proteins were detected using a 2-DE approach, which was found to be altered in relative abundance, while shotgun found variations in 118 proteins. Only 7% of DAPs were found to be common in both methods. The most affected metabolic pathways were signaling, protein metabolism, and oxidoreductases. Further findings suggested that Mn-toxicity mediated protein-turnover impaired the roots for energy production, leading to changes in oxidative phosphorylation, glycolysis, TCA, and pyruvate metabolism. Root proteome indicated a slowdown of metabolic activities, including call wall integrity, protein turnover, and energy production (Ceballos-Laita et al., 2018). Nevertheless, Mn interaction with Cd and attenuation of the toxic effects on plants are also found to play a major role in photosynthesis. Oliveira et al. (2020) studied the mechanisms of Mn-response in Cd toxicity mitigation in young plants of cacao by analyzing the alterations in DAPs and exclusive proteins (EP). Few significant proteins were produced in the presence of Cd while repressed in the presence of Cd + Mn, and vice versa. These findings suggested that Mn mitigated the adverse effects of Cd on cacao plants (Oliveira et al., 2020).

Two *Brassica napus* cultivars, ZS758 and ZD622, were treated with high As concentrations, and their response to As toxicity has been studied through iTRAQ-based proteomics analysis. The chlorophyll fluorescence attributes revealed that As pressure significantly lowered the photochemical efficiency of photosystem I and photosystem II and closed stomata detected under scanning electron microscopy. Metabolic pathways, followed by ribosome and secondary metabolites biosynthesis, were the dominant functional annotation among the differentially expressed proteins. Many genes involved in stress defense and primary metabolism were As-responsive DAPs (Farooq et al., 2021). Alka et al. (2021) performed histological and proteome analysis and found that *Microbacterium foliorum* lowered the As toxicity in *Melastoma malabathricum*. 2D gel electrophoresis and transmission electron microscopy were used to conduct the histological and proteome analysis. When As-treated cells were compared to untreated cells, considerable changes were discovered. Compared to control, root cells ultra-structure showed intact cell wall, cytoplasm, and vacuole under As + bacteria. To further understand the As + bacteria, proteome profiling of root cells was analyzed. It has been found that proteins involved in photosynthesis, defense, signaling, and protein biogenesis were higher in As + bacteria than As alone (Alka et al., 2021).

### Metabolomics

Metabolomics is considered as an emerging field that broadly detects and quantifies all exogenous and endogenous molecules of low molecular weight (<1 kDa), including metabolites present in living organisms (Razzaq et al., 2019; Raza, 2020). Multiple analytical techniques have been developed to understand plant metabolic responses like inductively joined MS, liquid, and gas chromatography-MS (LC-MS, GC-MS), and nuclear magnetic resonance spectroscopy (NMR) (Razzaq et al., 2019; Raza, 2020). Table 4 shows some examples of metabolomics studies under toxic metals/metalloids toxicity in different plants.

Xie et al. (2019) performed LC-MS/MS and HPLC analysis for metabolites and thiol compounds in *Amaranthus hypochondriacus* under Cd stress. *A. hypochondriacus* leaves accumulated Cd levels 40 times more than the control plant and also enhanced the PCs contents. Out of 12,084 metabolites identified, 41 were found to be significantly different metabolites (SDMs) among two groups and known to take part in seven different metabolic pathways. Among them, 12 SDMs related to PCs were associated with three different pathways, namely Arg and Pro metabolism, Val, Leu and Ile biosynthesis, and Ala, Asp, and Glu metabolism (Xie et al., 2019). An HPLC coupled with MS-based metabolome profiles of rice was analyzed in response to Cd and Cu toxicity. A total of 112 metabolites were identified, of which 97 metabolites were subsequently confirmed under Cd toxicity. Importantly, the secondary metabolism, amino acid metabolism-like purine, carbon, and glycerolipid metabolism pathways were found to be greatly affected. Furthermore, reduction in plant growth, photosynthetic capacity, and induction of defense systems to protect cell damage have also been observed (Navarro-Reig et al., 2017). In yet another study, GC-TOF-MS and LC-MS/MS-based metabolome profiling were performed to study the effect of Cu stress on cucumber plants (Zhao L. et al., 2018). Metabolomics helped in the identification of 149 primary and 79 secondary metabolites. Down-regulation of TCA intermediates (up to 1.4–2.4-fold) was observed, indicating disturbed carbohydrate metabolism. Excess Cu affected the aldarate and ascorbate metabolism and shikimate phenylpropanoid biosynthesis (Zhao L. et al., 2018). In soil, excess Se may appear destructive to plants. Zhang et al. (2019b) carried out an untargeted metabolome analysis of Se-treated and control celery seedlings and identified 24 sulfur and seleno-compound metabolic unigenes to be differentially expressed. Moreover, 1,774 metabolites and 237 SDMs were found through UHPLC-MS/MS. Results revealed that identified metabolites could be associated with significant biological pathways regulating Se tolerance (Zhang et al., 2019b). Furthermore, an untargeted metabolomics method was used to study the effects of 5 days application of 100 μmol L^–1^ selenate on broccoli sprouts metabolome. Multivariate statistical analysis displayed that tyrosine, D-erythronolactone, serine, and melezitose were up-regulated, while citric acid, D-glyceric acid, and succinic acid were down-regulated after selenate application. Selenate application also affected the metabolism of GSH, β-alanine, and plant-metabolite biosynthesis associated with glucosinolate precursors (Tian et al., 2018).

The metabolomic responses of tea plants toward Zn stress showed that Zn excess or deficiency differentially affected the metabolic pathways in tea leaves (Zhang et al., 2017). Zn-deficiency influenced carbohydrates metabolism, whereas Zn-excess affected the metabolism of flavonoids. Furthermore, it was observed that both Zn-excess and Zn-deficiency led to lowered nicotinamide concentrations, which accelerated NAD^+^ breakdown and resulted in low energy metabolism (Zhang et al., 2017). Furthermore, UHPLC/Q-TOF based metabolic investigation was conducted to study the effects of 100 mM NaCl and 100 mM ZnSO_4_ on lettuce root metabolic profiling (Rouphael et al., 2016). Most of the compounds identified in ZnSO_4_/NaCl environments were lipids, carbohydrates, glucosinolate, phenolics, or hormones. Results showed that osmotic stress and redox-imbalance have an essential role in defining lettuce root metabolic response; while polyamines and polyamine conjugates were found to be elicited as a precise reaction to ZnSO_4_ (Rouphael et al., 2016). *Qualea grandiflora* plants were grown in Murashige and Skoog (MS) medium with or without Al supplementation for 120 days. Metabolite profiling of *Qualea grandiflora* plants was performed through GC-MS under Al stress. *Q. grandiflora* plants with starved Al showed shorter roots and shoots, chlorotic leaves, and low biomass. Al was found to be critical for lignin synthesis, cell wall, processing of genetic information, and organic acid metabolism. Furthermore, Al was predicted to help plants uptake phosphorous (Cury et al., 2020).

Cosio and Renault (2020) studied physiology and shoot metabolomic profiles of *Elodea nuttallii* exposed to methyl-Hg (30 ng L^–1^), inorganic Hg (70 ng L^–1^), and Cd (280 μg L^–1^) for 24 h. KEGG pathway analysis revealed that MeHg exposure resulted in numerous biochemical changes like aminoacyl-tRNA biosynthesis, serine, glycine, threonine, proline, arginine, and nitrogen metabolism. In contrast, Cd-stress resulted in important alterations in aminoacyl-tRNA biosynthesis and branched-chain amino acid pathways. Data supported the argument that MeHg impacts N homeostasis, Cd caused an osmotic stress-like pattern, and inorganic Hg had less impact on both features (Cosio and Renault, 2020). In a different study, Pb-treated vetiver plants grown in a hydroponic setup were used for LC/MS/MS-based metabolome analysis of shoot, and root tissues. Multivariate metabolite analysis displayed tremendous induction of vital metabolic pathways like amino acid metabolism, sugar metabolism, enhanced osmoprotectants production like polyols and betaine, and metal chelating organic acids (Pidatala et al., 2016). For Pb remediation, vetiver grass is considered a superior choice due to its Pb-hyperaccumulation ability. Pidatala et al. (2018) performed comparative metabolic profiling of maize and vetiver under Pb-stress conditions. Vetiver plants displayed a massive increase of metabolites under Pb-stress, including coenzymes, amino acids, and organic acids; whereas, maize displayed a modest increase in the same metabolites with no significant effect on other metabolites (Pidatala et al., 2018). Furthermore, Zhang et al. (2019d) performed metabolic profiling of *Malus halliana* leaves under Fe-deficiency through GC/MS and identified a total of 18, 39, and 17 metabolites in three pairs L12h vs. L0h, L3d vs. L0h, and L3d vs. L12h, respectively. The findings showed that trehalose and sucrose are the most abundant metabolites in glucose metabolism and have a role in balancing photosynthetic activity in *M. halliana* leaves with the utilization of photo-assimilate. Overall, identified metabolites were found to play a major role in the positive regulation of Fe-deficiency response (Zhang et al., 2019d).

Das et al. (2021) investigated the differential expression of two *Andrographis paniculata* genotypes under As stress. The As distribution was found to be higher in roots than in other tissues with TF <1. APMS (wild collection) was more tolerant and accumulated less As than APwC (mass selection line). HPLC was used to quantify the metabolites in sample extract, and metabolites detection was performed at 223 nm by photodiode array detector (PDA). Arsenic enhanced flavonoids production like 5,7,2′,3′-tetramethoxyflavone in *A. paniculata*. The main secondary metabolites, oxidative enzymes, and nutrient uptake showed a significant difference in the detoxification process under As stress. The improved *Ap2* expression proposed its involvement in metabolic flux channeling toward the ent-LRDs biosynthesis under As stress (Das et al., 2021). NMR-based metabolomics approach provides a quick snapshot of metabolites without prior knowledge of organisms. Thus, Arora et al. (2018) validated the applicability of NMR-based metabolomics using freshwater microalga *Scenedesmus* sp. Using NMR spectroscopy, the authors identified and quantified around 45 metabolites, comprising organic acids, sugars, amino acids, nucleotides, phosphagens, and osmolytes. These findings revealed that microalga tolerated the As toxicity by accumulating these diverse metabolites (Arora et al., 2018).

### Ionomics

Plants require minerals in appropriate amounts to perform their functions properly. Ionome or ionomics are the mineral elements needed in trace amounts for plant growth, development, and removal of toxic metals/metalloids (Stich et al., 2020). Nitrogen is an essential plant nutrient and forms an integral part of nucleic acids, proteins, vitamins, and hormones. It reduces toxic metals/metalloids toxicity by synthesizing Chl, N-containing antioxidants, and metabolites (Lin T. et al., 2011). Punshon et al. (2013) used the ionomic approach of multi-element imaging synchrotron X-ray fluorescence microscopy (SXRF) with *A. thaliana* for studying the phenotype, gene identification, and screening. Su et al. (2017) performed Fourier transform infrared spectrometry (FTIR) analysis to examine the subcellular distribution, accumulation, alteration in metabolic activity, and physiology of *S. polyrhiza* under Cd stress (5, 10 μM for 4 days). In another study, ICP-MS ionomic approach was applied to sunflower growth under Cd stress (50, 350, and 750 mg as CdCl_2_). Increased doses of Cd caused necrosis and chlorosis and led to disproportion in Cu/Zn homeostasis (Júnior et al., 2014). Some recent examples of ionomics studies under toxic metals/metalloids toxicity are provided in Table 4.

Zhu et al. (2010) reported 16 mM N fertilizer (NH_4_)_2_SO_4_ to reduce the Cd toxicity in Sedum. In *S. nigrum*, the fertilizers, (NH_4_)_2_SO_4_ and CH_4_N_2_O application increased plant biomass by up to 2.0 and 2.1-folds, respectively, as compared to control under Cd stress (2 mg kg^–1^) (Yang et al., 2019). Mg, which is an essential component of Chl, reduced Cd toxicity by enhancing the activity of antioxidant enzymes (Chou et al., 2011). In Japanese mustard spinach, Cd stress and accumulation caused by a dose of 0.25 μM was reduced by up to 40% after the application of 10 mM Mg (Kashem and Kawai, 2007). Calcium not only regulates the metabolic activity of plants but also reduces the toxicity caused by toxic metals/metalloids. For instance, 30 mM Ca reduced Cd quantity from 46.7 to 17.4 μg in *A. thaliana* (Suzuki, 2005). Feng et al. (2017) performed inductively coupled plasma-MS (ICP-MS) determination of Cd/As to demonstrate a distinct transport of elements to 14 parts of 21 different brown rice genotypes. Interestingly, sixteen other elements were also determined in roots, and associated with the highest Cd accumulation fraction and increased glycolytic activity. Cu, Zn, Mg, and Co distribution was found to be associated with Cd concentration in roots and nodes. Ionomic profile indicated that all elements are correlated with different plant parts, and distribution occurs through them, while node1 had the highest accumulation rate of Cd (Feng et al., 2017).

The ionome of maize shoot has been studied under the Fe regime using recombinant inbred line population, and significant genotypic variance among 12 mineral elements was identified using inductively coupled plasma optical emission spectrometry (ICP−OES) (Stich et al., 2020). Besides, *ZmHMA2/3* and *ZmMOT1* were also proposed to cause genetic variation of Cd and Mo contents in maize shoots. Another ionome study targeting shoots and roots of *Brassica* species identified a total of 133 and 123 QTLs under optimal and low P (OP/LP) conditions (Wang et al., 2020e). These QTL clusters are predicted to reveal the uptake and transport mechanisms of mineral elements. Ionome study in rice seedlings, when mediated by nano-Si-priming (SiNP), generated fluoride tolerance by reducing fluoride uptake and bioaccumulation. This improved photosynthetic activity and uptake of mineral nutrients by lowering oxidative damage, thus ensuring a safe rice cultivation method (Banerjee et al., 2020). During 3 consecutive years, a decreasing trend of low contamination of toxic metals/metalloids was observed in an ionome study of potato tubers (Muntean et al., 2019). A study on rice grain and straw identified 70 novel ionomic QTLs for 15 nutrient elements. The study identified a molybdate transporter gene *OsMOT1;1* underlying *qMo8* QTL controlling Mo concentration in both grain and straw tissues (Wang et al., 2020a). Ionomic variations of rice had also been compared to *Arabidopsis* against sodium, molybdenum, and nitrogen using genome-wide association studies from 529 accessions. This comprehensive study with 6.4 million SNPs identified 72 loci for ionomic variation and further identified candidate genes, sodium transporter gene *Os-HKT1, Os-MOLYBDATE TRANSPORTER*, and thus grain number, plant height, and heading date thus featured the molecular mechanism controlling rice ionome (Yang et al., 2018). Mišúthová et al. (2021) studied the growth and developments of maize plants in response to silicon (Si) and As metalloids. The experimental findings revealed increased As (75 and 150 μM) concentration reduced the biomass and root length, and 2.5 μM Si supplementation to the medium did not affect root growth. Further, Si application did not affect the uptake of micro-and macro-elements into the roots (Ni, Ca, Zn, P, Cu, Mo, and K). While, Si significantly reduced the presence of hydrogen peroxide and superoxide in roots suffering from As toxicity and increased root antioxidant enzymes (Mišúthová et al., 2021). In another study, Xiao et al. (2021) investigated the transcriptomic variations and ionomic profiles to understand the molecular mechanism of alleviating the As(III) stress. Under As(III) stressed and control conditions, a total of 29 elements were found through inductively coupled plasma mass spectrometry (ICP-MS) in the rice roots and shoots. Strong connections were observed between transcriptome and ionome in the rice shoots. Overall, 3,812 DEGs were discovered in the As(III)-applied shoots than control shoots, and these DEGs were mainly associated with transmembrane transport and ion binding. The rice plants preferred to translocate more nutrients to cope with arsenic toxicity (Xiao et al., 2021).

### miRNAomics

miRNAs are tiny non-coding RNA species and key post-transcriptional regulators. They have also emerged as an important technique like other omics approaches to develop toxic metals/metalloids tolerant plants (Dubey et al., 2021; Jamla et al., 2021b). Some successful studies have been completed to identify metals/metalloids responsive miRNAs. For instance, miRNA against Cd toxicity have been reported in many crop plants, including wheat (Qiu et al., 2016), rice (Huang et al., 2009), maize (Gao et al., 2019), barley (Yu J. et al., 2019) and rapeseed (Jian et al., 2018). These successful studies depicted miRNAs regulating toxic metal uptake and transport, inducing oxidative stress and antioxidative defense, thus maintaining homeostasis. The exploration of miRNAs in plants, along with other omics approaches and computational tools, may act as potential targets for improving plants to confer toxic metals/metalloids tolerance (Dubey et al., 2021). Some recent reports of miRNAs against toxic metals/metalloids stress and their impact on plants are mentioned below.

The role of *miR156j* from *miR156* family was observed in rice for physiological growth stages against As toxicity. Expression analysis revealed the functional role of *Osa-miR156j* at various developmental stages and plant tissues under As(V) exposure. Further, stress-responsive *cis*-acting regulatory elements confirmed its involvement during stress conditions. The results altogether showed the obvious role of *miR156j* in metabolic activities under As toxicity and can be used to develop As stress-tolerant varieties (Pandey et al., 2020). The genome-wide identification analysis in *Brassica juncea* identified some novel miRNAs, including 11 miRNAs and 56 transcripts against Cd stress response, using transcriptomic and sRNA sequencing technology. This study found *bra-miR172b-3p* regulating *ATCCS* playing a significant role against Cd stress and highlighted the regulatory relationship between miRNAs and transcripts, thus providing insights into molecular mechanisms in response to Cd stress in plants (Liu et al., 2021). A library of more than 2,000 artificial microRNAs (amiRNAs) has been developed in *Arabidopsis* to overcome the limitation for dissecting As responsive mechanisms. The research identified six transformant lines in response to As(V) and Cd exposure. However, further characterization of miRNA line closely related to homologous CBF and ERF transcriptional factor genes revealed *ERF34* and *ERF35* as important resistant TFs against Cd stress. The CBF1, CBF2, and CBF3 TFs were mediating negative regulation under As exposure and can be further used in the future to identify Cd and As induced transcriptional control networks (Xie et al., 2021).

Another miRNA named *miR1511* for the *MIR1511* gene responded against Al toxicity in common bean and then targeted against *ALS3* (*Aluminum Sensitive Protein 3*) gene. Both the genes opposed each other for sensitive genotypes as decreased expression of *miR1511* showed increased *ALS3* transcript level under Al toxicity and vice versa, thus revealing the prominent role of *miR1511* for inducing resistance against Al stress in plants (Ángel et al., 2021). Overexpression of *miR156* had significantly reduced the endogenous ROS in *Arabidopsis* and enhanced tolerance against Cd stress by reducing the effect of Cd-related transporters (Zhang et al., 2020c). In *Miscanthus sinensis*, high-throughput miRNA sequencing for Cr toxicity identified a total of 104 conserved miRNAs and 158 non-conserved miRNAs, out of which 45 differentially expressed miRNAs were linked with roots while 13 differentially expressed miRNAs were linked with leaves under Cr stress. GO, and KEGG analysis identified *miR167a, novel-miR15*, and *novel-miR22* as potential candidates that were involved in Cr transportation and chelation. Moreover, *miR156a, miR164, miR396d*, and novel *miR155* were involved in physiological and biochemical metabolisms and detoxifying the toxic effect of Cr in plants (Nie et al., 2021). Overall, these studies showed that miRNA significantly regulates genes expression and plays a key role in mitigating the adverse effect of toxic metals/metalloids. Thus, miRNAomics should be more exploited in identifying tiny key players (miRNAs) in regulating the stress-responsive mechanisms against a variety of toxic metals in different crop plants.

### Phenomics

A phenome is a set of all biological, physical, and biochemical processes expressed by an organism in the form of phenotypes (qualitative and quantitative features) in a specific living condition. Plant phenomics depicts the phenotypic and genotypic expression within a particular living condition (Pratap et al., 2019). The phenotype of a plant may have a diverse manifestation within the same genotype due to different environmental conditions, i.e., abiotic stresses. The plant phenotype can be identified by using accomplishments made via genomic innovations and bioinformatics with the application of analytical techniques/phenomics technologies determining the relationship between plant genotype and environmental stress (Walter et al., 2015). The significance of phenomics is highly appreciable in the post-genomic era and is noticeable after using approaches like genomic selection, marker-assisted selection, GWAS, and QTL mapping, which are highly dependent on HTP for crop improvement. HTP technologies collect large data samples with automated digital analysis, interpret and process the data accurately with adequate statistical processing (Awada et al., 2018). The use of recent approaches for genetic dissection, knowledge of plant structure, and investigation of all functional processes enable the identification of plant phenotypic expression (Pratap et al., 2019). Some reported and widely used tools for phenotyping plants are provided in Supplementary Table 1. The integration of phenomics with other omics approaches forms the basis of crop breeding to identify plants with the best acceptable phenotype. It is the most promising approach for achieving sustainability in agriculture by understanding the plant’s response to multiple stresses, including toxic metals/metalloids toxicity.

Recently, root system architecture (RSA) traits have been studied in soybean using the phenome approach to identify genetic diversity since it is difficult to analyze RSA traits manually for a large number of accessions (Falk et al., 2020). Genotype and phenotype-based clusters of accessions showed similarity, in which genotype-based clusters depicted co-relation by geographical origins. This analysis provides opportunities for the breeding of root traits using beneficial functional genetic diversity that can be used in the future for crop improvements (Falk et al., 2020). In *A. thaliana*, a dataset of stress-responsive signals has been developed under a wide range of biotic and abiotic stresses, including Al and Fe toxicity, temperature, light, osmotic, and oxidative stresses. This research highlighted several shared and unshared biological processes, molecular functions, metabolic pathways, and phenomic characteristics that may help develop improved varieties using genome editing techniques (Naika et al., 2013). Since minimal applications of phenomics are reported against toxic metals/metalloids toxicity in plants, there is a margin to fulfill this gap by utilizing the phenomics approach to develop tolerant varieties by breeding crop plants against metals/metalloids toxicity.

## Online Databases/Tools for Effective Integration of Omics Data

The diversity and large volumes of omics data present a need for various tools required for data analysis. Several omics platforms have been developed to meet the needs and to integrate better the generated multi-omics data (Hernández-de-Diego et al., 2018). These tools are useful to decipher plant responses and behavior through results from the varying levels of molecular information. A few selected databases and tools/software for effective integration of the multi-omics data are discussed in this section. Some other examples, along with their functionality, have been provided in Supplementary Table 2.

One of the web databases, the Plant Omics Data Centre (PODC), allows gene mining by acquiring publicly available transcriptomics data obtained through RNA-sequencing of different model and crop plants. It also comprehensively integrates the gene expression networks and provides some essential features on inter species diversity by comparing gene expression networks, manual addition of information on published genes, and functional gene annotation through text mining (Shenton et al., 2019). Another web database, CATchUP, deals with the Spatio-temporal gene expression, i.e., genes expressed under specific conditions, by identification of the maximum expression difference of the transcripts. Owing to its unique data mining properties under particular treatment/conditions, it is mainly useful to find tissue-specific genes expressed at a certain developmental stage (Shenton et al., 2019). The National Genomics Data Centre (NGDC) processes a wide range of genomics-related data available through a family of database resources. This suite of databases has allowed data integration and curation, which can be accessed in either of three categories of resources, namely meta and sequenced data, standardized information, and processed data. Some prominent databases maintained by NGDC include manually curated Genome-Wide Variant-Trait Association (GWAS) Atlas, Genome Warehouse (GWH), Genome Variation Map (GVM), Plant Editosome Database (PED), and The Methylation Bank/MethBank (National Genomics Data Center Members and Partners, 2020).

Studies have shown that different tools and techniques involving joint visualization of the multi-omics data provide molecular context and a better understanding of the molecular interconnections (Hernández-de-Diego et al., 2018). One of the metabolic pathway-based data visualization tools, KaPPa-View, allows the integration of transcript and metabolite data into pathway maps (Hernández-de-Diego et al., 2018) and hence helps to find a correlation with the biological knowledge. Similarly, a protein classification and annotation framework, MapMan4, supports multiple omics platforms, including transcriptomics, proteomics, and metabolomics (Schwacke et al., 2019). Another web-based tool, PaintOmics3, lays a framework for interactive exploration of multi-omics data via the KEGG pathway diagram and has enabled researchers to understand different levels of regulation in a biological system (Hernández-de-Diego et al., 2018). MetaBridge is a database that uses data from KEGG and MetaCyc metabolic pathway databases. It is useful for data integration and gene mapping for specific metabolites (Blimkie et al., 2020). The Miodin (MultI-Omics Data INtegration) R package allows users to integrate the omics data in a streamlined workflow-based syntax either based on vertical (across experiments on the same samples) or horizontal (across studies on the same variables) integration of samples. It supports the multi-omics modalities and enables workflows to import efficiently, process, and analyze the multi-omics data (Ulfenborg, 2019).

Besides the fact that these tools and databases have proved useful for researchers, the fast-evolving multi-omics platforms have resulted in the lack of uniformity among the available tools. This necessitates committed efforts for quality assessment and validation from scientific communities across the world.

## An Overview of Plant-Based Remediation Approaches for Toxic Metals/Metalloids

Rapid industrialization has resulted in a large number of toxic metals/metalloids pollutants into the soil, water, and air, which then enter crops directly or indirectly through various pathways, posing significant health and wildlife risks (Rai et al., 2019; Raza et al., 2020, 2021a). Nevertheless, different plant-based remediation techniques have been developed to solve these issues, and some of the major techniques have been briefly discussed in the subsequent sections.

### Bioremediation

Bioremediation, particularly biochar amended phytoremediation, is one of the most promising remediation strategies that is widely used to remediate toxic metals/metalloids in the soil (Shukla A. et al., 2018; Wang et al., 2021). Biochar has many significant properties that make it a good remediation material for contaminated soils, including a large internal surface area, negative charge, and resistance to degradation. It is also the most important bioremediation strategy since it not only serves to remediate soil pollution and improve soil quality but is also a cost-effective product that decreases the global environmental challenge of solid waste management (Shukla A. et al., 2018; Wang et al., 2021). A recent study shows that the addition of 10% of biochar generated from rice straw to Cu-contaminated soil reduced Cu availability by 96% (Rehman et al., 2019). Furthermore, biochar made from rice husk pyrolysis was found to be a useful agricultural by-product and an excellent immobilizer of metals contaminants such as Zn, Pb, and Cd (Dhiman et al., 2021). Sulfur-modified rice husk biochar advanced its sulfur content and was utilized as a “green bioremediation approach” to stabilize Hg-polluted soil (O’Connor et al., 2018). Metal pollutants are taken in and concentrated by hyperaccumulators and organic soil remedies, resulting in their precipitation within the root’s innards (Huang et al., 2018). *Cassia alata*, a promising photo stabilizer with biochar aid to improve soil pH, nitrogen content, and total carbon, reduces Pb and Cu metal contamination in mine tailings and also leads to increased plant shoot and root biomass (Huang et al., 2018).

### Phytostabilization

Phytostabilization is the process in which plant species with high metal tolerance are used to immobilize toxic metals/metalloids below ground and reduce their bioavailability, thus preventing metals from migrating into the environment and reducing the probability of metals/metalloids entering the food chain (Raza et al., 2020; Yan et al., 2020). In this approach, toxic metals/metalloids are physically and chemically immobilized in the rhizosphere through sorption on roots of plants, fixation with soil amendments, complexation, precipitation, or reduction in metals valence (Mthembu et al., 2020). Phytostabilization requires the identification of appropriate plant species with high resistance to toxic metals/metalloids (Yan et al., 2020). Because of their better tolerance to toxic metals/metalloids, territorial expansion, and evapotranspiration, some plant species, particularly crop grasses and willows (*Salix* spp.), are effective for phytostabilization (Sylvain et al., 2016). Plants such as *Festuca* spp. and *Agrostis* spp. are commonly used to phytostabilize Pb, Zn, and Cu contaminants in soil (Galende et al., 2014). Lands with increased metals/metalloids contamination or physical disturbances lack natural flora; hence, phytostabilization techniques can be used to rebuild a vegetative cover. Wind erosion of surface soils and leaching to groundwater is reduced when metal-tolerant plants are planted in such areas (Mthembu et al., 2020).

### Phytoextraction

Phytoextraction is the process of absorbing, transporting, and accumulating toxic metals/metalloids in the biomass of harvestable plant parts using specialized and highly adapted hyperaccumulators (Diarra et al., 2021). The electrochemical characteristics of the metals being extracted, as well as their absorption, transport, and accumulation in the plants, all influence the efficiency of the phytoextraction process (Saini and Dhania, 2020). The most important factor determining the viability and success of phytoextraction is plant selection criteria; therefore, hyperaccumulators with exceptional tolerance and bioaccumulation of a broad range of toxic metals/metalloids are preferred (Diarra et al., 2021). Over 500 plant species from 45 different families, including Lamiaceae, Caryophyllaceae, Poaceae, Asteraceae, Fabaceae, and Brassicaceae, have been identified as hyperaccumulators so far. Brassicaceae is the most common family among those identified (Gul et al., 2021). In a recent study, the flowering plant *Lantana camara* L. exhibited all of the desired criteria of a hyperaccumulator plant, including a translocation factor (TF) >1 and Cd content in aerial portions of >100 mg kg^–1^. As a result, it was proposed as a Cd hyperaccumulator (Liu S. et al., 2019). *Artemisia vulgaris* was recently identified as a promising plant for metal phytoremediation, with TF > 11 for As, Cd, Cu, Cr, Ni, and Pb, while *Silene vulgaris* had a TF greater than 1 for Cd (Antoniadis et al., 2020).

## Conclusion and Future Prospects

Climate change and agricultural production are highly correlated, as climate change is the leading cause of several environmental stresses, including toxic metals/metalloids toxicity in the soil and environment. Increasing metals/metalloids toxicity poses a severe threat to agricultural production by hampering plant growth and yield. The toxic effects of these toxic metals/metalloids also significantly affect the plant’s physiological, biochemical, and molecular mechanisms, which are vital for healthy plant growth and improved yield. Several plant-based remediation approaches have also been successfully used to remediate the toxicity of the toxic metals/metalloids in the soil and environment. Under metals/metalloids toxicity, plants adjust themselves by modifying genes, proteins, and metabolites expression levels to cope with unfavorable conditions. Thus, a comprehensive understanding of the biological processes is required to cope with toxic metals/metalloids toxicity in the emerging technological era. During the last few years, significant progress has been made in utilizing state-of-the-art “omics” approaches to develop climate-smart plants. Interestingly, genome-wide omics analysis holds the potential to identify stress-related genes, metabolites, proteins, minor and major elements, miRNAs, stress regulators, and metabolic pathways precisely associated with plant phenotype under stressful environments.

**FIGURE 3:**
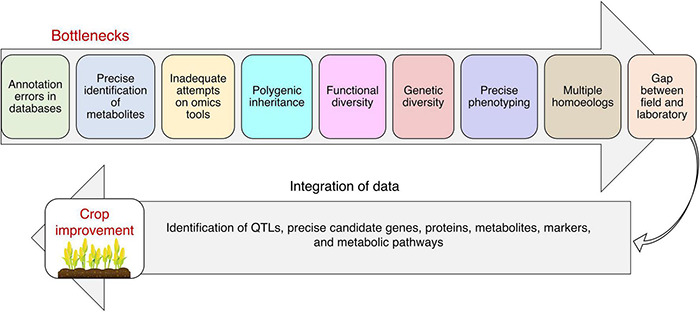
Persisting bottlenecks in the exploitation of omics approaches (Raza et al., 2021b). Although an outstanding revolution has been accomplished in the biotechnological era, many queries and bottlenecks presently limit the utilization of omics approaches for stress tolerance research in several plant species. The amendment of these bottlenecks using molecular tools will help us to exploit the innovative manifesto in developing toxic metals/metalloids tolerant plants.

Nevertheless, some persisting bottlenecks in exploiting omics approaches demand urgent attention (Figure 3; Raza et al., 2021b,d). Polygenic inheritance and genetic diversity are significant bottlenecks among the existing ones. Therefore, releasing these bottlenecks using molecular tools will help us to feat the new strategy to develop stress resistance plants; and consecutively will guarantee global food safety. For instance, recent developments in high-throughput technologies enable the analysis of global genetic diversity. Genomic selection and haplotype-based breeding are expected to lessen the genetic diversity of a breeding plan in the long term. Consequently, sustaining genetic diversity in breeding programs will be essential for maintaining genetic gains from breeding revolutions (Varshney et al., 2021a,b, c). Therefore, integrating ideal selection contributions with these methodologies may help preserve genetic diversity while enhancing genetic gains. Furthermore, effective crop breeding programs with an improved genetic base will speed up fast-forward breeding, proposed by Varshney et al. (2021b). Briefly, genomics-assisted breeding, including haplotype-GWAS, haplotype-assisted breeding, and haplotype-assisted genomic selection, will effectively utilize superior haplotypes to accelerate future breeding advancement (Bhat et al., 2021). Moreover, modern machine learning tools and artificial intelligence methodologies systematically incorporate the overflow of data rolling through multi-omics approaches (van Dijk et al., 2020; Jung et al., 2021). Hence, system-level knowledge will help clarify functional diversity and regulatory networks underlying intricate phenotypes of agricultural value.

Genome editing by CRISPR/Cas systems and genetic engineering of stress-associated genes could be essential prospects for improving stress tolerance. Likewise, the engineering of metabolic pathways could also provide new avenues for the development of climate-resilient plants. Recently, speed breeding has emerged as the most powerful and time-saving tool to shorten the breeding cycle and enhance genetic gains in plants. Therefore, omics approaches could be galvanized by utilizing speed breeding to facilitate plant breeders to keep pace with increasing environmental fluctuations and burgeoning human population.

## Author Contributions

AR conceived the idea and prepared the figures. AR, JT, ZZ, SC, SB, RB, CZ, and HC contributed in writing. AR, JT, ZZ, and SC designed the tables. AR, RB, FBJ, RSAK, RKV, and WZ reviewed and edited the manuscript. All authors have read and approved the final version of the manuscript.

## Conflict of Interest

The authors declare that the research was conducted in the absence of any commercial or financial relationships that could be construed as a potential conflict of interest.

## Publisher’s Note

All claims expressed in this article are solely those of the authors and do not necessarily represent those of their affiliated organizations, or those of the publisher, the editors and the reviewers. Any product that may be evaluated in this article, or claim that may be made by its manufacturer, is not guaranteed or endorsed by the publisher.
