# Supplementary material for: Advances in “Omics” Approaches for Improving Toxic Metals/Metalloids Tolerance in Plants
Source: Front Plant Sci. 2022 Jan 4;12:794373. doi: 10.3389/fpls.2021.794373 (PMC8764127; doi:10.3389/fpls.2021.794373)
Supplement: Supplementary file 1 [file Data_Sheet_1.docx]

**Supplementary Table 1.** Tabular account of some available phenomics tools for plant investigations under stressful conditions

| **Tool** | **Specification** | **URL** | **Provider** | **Reference** |
| --- | --- | --- | --- | --- |
| Wiwam-Automated Systems For Plant Phenotyping | Tool for automated imaging and precise irrigation of plants | <https://www.wiwam.be/> | SMO and VIB  (Flanders Institute for Biotechnology), Belgium | Asaari et al. (2019) |
| North American Plant Phenotyping Network (NAPPN) | Quantify phenotypic data | <http://nappn.plant-phenotyping.org/> | McGill University, Canada  Washington State University, USA | Carroll et al. (2019) |
| Australian Plant Phenomics Facility (APPF) | Phenotyping, data analysis through bioinformatics and biometry | <https://www.plantphenomics.org.au/> | University of Adelaide;  Australian National University;  CSIRO Agriculture and Food, Australia | Borjigin et al. (2020) |
| Plant Phenomics Research Centre (PPRC) | Phenotyping, high-throughput monitoring | <http://pprc.njau.edu.cn/> | Nanjing Agricultural University, China | Colmer et al. (2020) |
| EMPHASIS | Phenotyping, data and computational service | <https://emphasis.plant-phenotyping.eu/> | Institute for Plant Sciences, Germany | Papoutsoglou et al. 2020) |
| European Plant Phenotyping Network (EPPN) | Develop sensor technology, IT standards for plant phenotyping | <https://www.plant-phenotyping-network.eu/> | Institute of Bio- and Geosciences IBG-2: Plant Sciences, Germany | Nagel et al. (2020) |
| PhenomUK | Crop phenotyping, technological innovation, 3D reconstruction, bioimage analysis | <https://www.phenomuk.net/> | University of Nottingham, UK | N/A |
| International Plant Phenotyping Network  (IPPN) | Good phenotyping, IT for phenotyping, novel instrumentation | <https://www.plant-phenotyping.org/> | Nanjing Agricultural University, China; University of Adelaide, Australia; KAUST  Saudia Arabia | N/A |

**References**

Asaari, M.S.M., Mertens, S., Dhondt, S., Inzé, D., Wuyts, N., Scheunders, P. (2019). Analysis of hyperspectral images for detection of drought stress and recovery in maize plants in a high-throughput phenotyping platform. *Comput. Electron. Agric.* 162, 749-758.

Borjigin, C., Schilling, R.K., Bose, J., Hrmova, M., Qiu, J., Wege, S., Situmorang, A., Brien, C., Berger, B., Gilliham, M. (2020). A single nucleotide substitution in TaHKT1; 5-D controls shoot Na+ accumulation in bread wheat. *bioRxiv* doi:10.1101/2020.01.21.909887.

Carroll, A.A., Clarke, J., Fahlgren, N., Gehan, M.A., Lawrence-Dill, C.J., Lorence, A. (2019). NAPPN: Who we are, where we are going, and why you should join us! *Plant Phenome J.* 2, 1-4.

Colmer, J., O’Neill, C.M., Wells, R., Bostrom, A., Reynolds, D., Websdale, D., Shiralagi, G., Lu, W., Lou, Q., Le Cornu, T. (2020). SeedGerm: a cost‐effective phenotyping platform for automated seed imaging and machine‐learning based phenotypic analysis of crop seed germination. *New. Phytol.* 228, 778-793.

Nagel, K.A., Lenz, H., Kastenholz, B., Gilmer, F., Averesch, A., Putz, A., Heinz, K., Fischbach, A., Scharr, H., Fiorani, F. (2020). The platform GrowScreen-Agar enables identification of phenotypic diversity in root and shoot growth traits of agar grown plants. *Plant Methods* 16, 1-17.

Papoutsoglou, E.A., Faria, D., Arend, D., Arnaud, E., Athanasiadis, I.N., Chaves, I., Coppens, F., Cornut, G., Costa, B.V., Ćwiek‐Kupczyńska, H. (2020). Enabling reusability of plant phenomic datasets with MIAPPE 1.1. *New Phytol.* 227, 260-273.
