# Supplementary material for: Advances in “Omics” Approaches for Improving Toxic Metals/Metalloids Tolerance in Plants
Source: Front Plant Sci. 2022 Jan 4;12:794373. doi: 10.3389/fpls.2021.794373 (PMC8764127; doi:10.3389/fpls.2021.794373)
Supplement: Supplementary file 2 [file Data_Sheet_2.docx]

**Supplementary Table 2.** List of tools and databases for integration of multi-omics data in plants

| **Tools and databases** | | **Omics integration** | | **Functionality** | **Future interventions/developments** | **URL** | | **References** |
| --- | --- | --- | --- | --- | --- | --- | --- | --- |
| **Databases** | | | | | | | | |
| KaPPa-View4 | | Metabolomics and Transcriptomics | | Represents transcriptomics data on metabolic pathway maps | Automated update of data on KaPPA-View4 KEGG. | <http://kpv.kazusa.or.jp/kpv4/> | | Sakurai et al. (2011) |
| MADMAX | Genomics, Metabolomics, and Transcriptomics | | Stores and analyzes multi-omics datasets | | Gene expression profiling and improvement of functional annotation of genes. | <http://madmax2.bioinformatics.nl/> | | Lin et al. (2011) |
| Mix Omics | Metabolomics, Metagenomics, Proteomics, and Transcriptomics | | Data exploration and visualization,  Dimensionality reduction | | NP-integration to integrate large scale data. | <http://mixomics.org/> | | Rohart et al. (2017) |
| MetaboAnalyst 4.0 | Metabolomics, Metagenomics, and Transcriptomics | | Data processing and visualization  Statistical analysis and functional interpretation | | Integration of metabolic sets and pathway libraries of model organisms other than humans.  Pathway analysis module to be updated to support interactive visual analysis. | <https://www.metaboanalyst.ca/> | | Chong et al. (2018) |
| Plant Metabolic Network | Genomics, Metabolomics, and Proteomics | | Plants specific databases containing pathways, enzymes, reactions, and compounds | | Improvement in enzyme functional annotation  Integration of physical chromosomal span, evolutionary patterns, protein-protein interactions, epigenetic modification marks, and biochemical reactions to better predict plant metabolic gene clusters. | <https://plantcyc.org/> | | Pinu et al. (2019) |
| **Tools/Software** | | | | | | | | |
| **Name** | **Functionality** | | | | | **URL** | | **References** |
| MONGKIE | Enables network analysis and visual mining of multi-omics data. | | | | | <http://yjjang.github.io/mongkie> | | Jang et al. (2016) |
| Pathview | Carries out pathway-based data integration and visualization. | | | | | <https://pathview.uncc.edu> | | Luo et al. (2017) |
| SLIDE | Performs the feature-level and group-level data visualization and allows independent analysis through the creation of customized gene lists. | | | | | <https://github.com/soumitag/SLIDE> | | Ghosh et al. (2019) |
| MapMan | Data visualization and comparative gene expression. | | | | | <https://mapman.gabipd.org/> | | Schwacke et al. (2019) |
| MetaBridge | Maps the metabolite data to perform pathway visualization and functional analysis with other omics data. | | | | | <https://metabridge.org> | | Blimkie et al. (2020) |
| IMPaLA | Integrates the pathway knowledge from databases and performs enrichment analysis with metabolite data. | | | | | [http://impala.molgen.mpg.de](http://impala.molgen.mpg.de/) | | Canzler et al. (2020) |
| Machado | Provides a framework to store, browse, and visualize biological data. | | | | | <https://github.com/lmb-embrapa/machado> | | Mudadu and Zerlotini (2020) |
| **Databases** | | | | | | | | |
| MOPED | | Provides information on the protein absolute and relative expression data along with gene relative expression data. | | | | | [http://moped.proteinspire.org](http://moped.proteinspire.org/) | Montague et al. (2014) |
| MODEM | | Allows genetic mapping and multi-dimensional omics data integration and visualization. | | | | | <http://modem.hzau.edu.cn> | Liu et al. (2016) |
| MetaCyc/BioCyc | | Provides information on metabolic pathways and enzymes. | | | | | <https://metacyc.org/>  <https://biocyc.org/> | Caspi et al. (2016) |
| HMOD | | Provides a comprehensive set of omics data and KEGG pathway information for herbal medicine plants. | | | | | <http://herbalplant.ynau.edu.cn> | Wang et al. (2018) |
| KEGG | | An integrated database that provides information on chemicals, genomes, and systemic functional biological pathways. | | | | | <https://www.kegg.jp> | Kanehisa et al. (2019) |
| MaGenDB | | Integrates functional annotations at gene, transcript, and protein levels. | | | | | <http://magen.whu.edu.cn> | Wang et al. (2020) |
| Coriander Genomics Database | | Allows the systematic comparative and evolutionary analyses through cross-species collinearity. | | | | | <http://cgdb.bio2db.com> | Song et al. (2020a) |
| ZEAMAP | | Provides information on genes and comparative expression patterns. | | | | | <http://www.zeamap.com> | Gui et al. (2020) |

**References**

Blimkie, T., Lee, A.H.Y., Hancock, R.E. (2020). MetaBridge: An Integrative Multi‐Omics Tool for Metabolite‐Enzyme Mapping. *Curr. Protoc. Bioinform.* 70, e98.

Canzler, S., Schor, J., Busch, W., Schubert, K., Rolle-Kampczyk, U.E., Seitz, H., Kamp, H., von Bergen, M., Buesen, R., Hackermüller, J. (2020). Prospects and challenges of multi-omics data integration in toxicology. *Arch. Toxicol.* 94, 371–388.

Caspi, R., Billington, R., Ferrer, L., Foerster, H., Fulcher, C.A., Keseler, I.M., Kothari, A., Krummenacker, M., Latendresse, M., Mueller, L.A. (2016). The MetaCyc database of metabolic pathways and enzymes and the BioCyc collection of pathway/genome databases. *Nucl. Acids Res.* 44, D471-D480.

Chong, J., Soufan, O., Li, C., Caraus, I., Li, S., Bourque, G., Wishart, D.S., Xia, J. (2018). MetaboAnalyst 4.0: towards more transparent and integrative metabolomics analysis. *Nucl. Acids Res.* 46, W486-W494.

Ghosh, S., Datta, A., Tan, K., Choi, H. (2019). SLIDE–a web-based tool for interactive visualization of large-scale–omics data. *Bioinformatics* 35, 346-348.

Gui, S., Yang, L., Li, J., Luo, J., Xu, X., Yuan, J., Chen, L., Li, W., Yang, X., Wu, S. (2020). ZEAMAP, a comprehensive database adapted to the maize multi-omics era. *iScience* 23, 101241.

Jang, Y., Yu, N., Seo, J., Kim, S., Lee, S. (2016) MONGKIE: an integrated tool for network analysis and visualization for multi-omics data. *Biol. Direct* 11, 1-9.

Kanehisa, M., Sato, Y., Furumichi, M., Morishima, K., Tanabe, M. (2019). New approach for understanding genome variations in KEGG. *Nucl. Acids Res.* 47, D590-D595.

Lin, K., Kools, H., de Groot, P.J., Gavai, A.K., Basnet, R.K., Cheng, F., Wu, J., Wang, X., Lommen, A., Hooiveld, G.J. (2011a) MADMAX–Management and analysis database for multiple~ omics experiments. *J. Integr. Bioinfor.* 8, 59-74.

Liu, H., Wang, F., Xiao, Y., Tian, Z., Wen, W., Zhang, X., Chen, X., Liu, N., Li, W., Liu, L. (2016). MODEM: multi-omics data envelopment and mining in maize. *Database* 2016, baw117.

Luo, W., Pant, G., Bhavnasi, Y.K., Blanchard Jr, S.G., Brouwer, C. (2017). Pathview Web: user friendly pathway visualization and data integration. *Nucl. Acids Res.* 45, W501-W508.

Montague, E., Stanberry, L., Higdon, R., Janko, I., Lee, E., Anderson, N., Choiniere, J., Stewart, E., Yandl, G., Broomall, W. (2014). MOPED 2.5—an integrated multi-omics resource: multi-omics profiling expression database now includes transcriptomics data. *Omics: A. J. Integr. Biol.* 18, 335-343.

Mudadu, M. D. A., Zerlotini, A. (2020). Machado: open source genomics data integration framework. GigaScience, 9(9), giaa097.

Pinu, F.R., Beale, D.J., Paten, A.M., Kouremenos, K., Swarup, S., Schirra, H.J., Wishart, D. (2019). Systems biology and multi-omics integration: Viewpoints from the metabolomics research community. *Metabolites* 9, 76.

Rohart, F., Gautier, B., Singh, A., Lê Cao, K-A. (2017). mixOmics: An R package for ‘omics feature selection and multiple data integration. *PLoS Comput. Biol.* 13, e1005752.

Sakurai, N., Ara, T., Ogata, Y., Sano, R., Ohno, T., Sugiyama, K., Hiruta, A., Yamazaki, K., Yano, K., Aoki, K. (2011). KaPPA-View4: a metabolic pathway database for representation and analysis of correlation networks of gene co-expression and metabolite co-accumulation and omics data. *Nucl. Acids Res*. 39, D677-D684.

Schwacke, R., Ponce-Soto, G.Y., Krause, K., Bolger, A.M., Arsova, B., Hallab, A., Gruden, K., Stitt, M., Bolger, M.E., Usadel, B. (2019). MapMan4: a refined protein classification and annotation framework applicable to multi-omics data analysis. *Mol. Plant.* 12, 879-892.

Song, X., Nie, F., Chen, W., Ma, X., Gong, K., Yang, Q., Wang, J., Li, N., Sun, P., Pei, Q. (2020a). Coriander Genomics Database: a genomic, transcriptomic, and metabolic database for coriander. *Hortic. Res.* 7, 1-10.

Wang, D., Fan, W., Guo, X., Wu, K., Zhou, S., Chen, Z., Li, D., Wang, K., Zhu, Y., Zhou, Y. (2020). MaGenDB: a functional genomics hub for *Malvaceae* plants. *Nucl. Acids Res.* 48, D1076-D1084.

Wang, X., Zhang, J., He, S., Gao, Y., Ma, X., Gao, Y., Zhang, G., Kui, L., Wang, W., Wang, Y. (2018). HMOD: an omics database for herbal medicine plants. *Mol. Plant* 11, 757-759.
